# The Triple Combination of Meropenem, Avibactam, and a Metallo-β-Lactamase Inhibitor Optimizes Antibacterial Coverage Against Different β-Lactamase Producers

**DOI:** 10.1016/j.eng.2024.02.010

**Published:** 2024-07

**Authors:** Zhuoren Ling, Alistair James Macdonald Farley, Aditya Lankapalli, Yanfang Zhang, Shonnette Premchand-Branker, Kate Cook, Andrei Baran, Charlotte Gray-Hammerton, Claudia Orbegozo Rubio, Edgars Suna, Jordan Mathias, Jürgen Brem, Kirsty Sands, Maria Nieto-Rosado, Maria Mykolaivna Trush, Nadira Naznin Rakhi, Willames Martins, Yuqing Zhou, Christopher Joseph Schofield, Timothy Walsh

**Affiliations:** a Department of Biology & Ineos Oxford Institute for Antimicrobial Research, University of Oxford, Oxford OX1 3RE, UK; b Chemistry Research Laboratory, Department of Chemistry and the Ineos Oxford Institute for Antimicrobial Research, University of Oxford, Oxford OX1 3TA, UK; c Latvian Institute of Organic Synthesis, Riga LV-1006, Latvia; d Department of Medical Microbiology, Division of Infection and Immunity, School of Medicine, Cardiff University, Cardiff CF14 4XN, UK; e Enzymology and Applied Biocatalysis Research Center, Faculty of Chemistry and Chemical Engineering, Babes-Bolyai University, Cluj-Napoca 400028, Romania

**Keywords:** Carbapenemase, Metallo/serine-β-lactamase inhibitor, Avibactam, Meropenem, Antimicrobial resistance

## Abstract

This work explores the potential of a triple combination of meropenem (MEM), a novel metallo-β-lactamase (MBL) inhibitor (indole-2-carboxylate 58 (InC58)), and a serine-β-lactamase (SBL) inhibitor (avibactam (AVI)) for broad-spectrum activity against carbapenemase-producing bacteria. A diverse panel comprising MBL- and SBL-producing strains was used for susceptibility testing of the triple combination using the agar dilution method. The frequency of resistance (FoR) to MEM combined with InC58 was investigated. Mutants were sequenced and tested for cross resistance, fitness, and the stability of the resistance phenotype. Compared with the double combinations of MEM plus an SBL or MBL inhibitor, the triple combination extended the spectrum of activity to most of the isolates bearing SBLs (oxacillinase-48 (OXA-48) and *Klebsiella pneumoniae* carbapenemase-2 (KPC-2)) and MBLs (New Delhi metallo-β-lactamases (NDMs)), although it was not effective against Verona integron-encoded metallo-β-lactamase (VIM)-carrying *Pseudomonas aeruginosa* (*P. aeruginosa*) and OXA-23-carrying *Acinetobacter baumannii* (*A. baumannii*). The FoR to MEM plus InC58 ranged from 2.22 × 10^−7^ to 1.13 × 10^−6^. The resistance correlated with mutations to *ompC* and *comR*, affecting porin C and copper permeability, respectively. The mutants manifested a fitness cost, a decreased level of resistance during passage without antibiotic pressure, and cross resistance to another carbapenem (imipenem) and a β-lactamase inhibitor (taniborbactam). In conclusion, compared with the dual combinations, the triple combination of MEM with InC58 and AVI showed a much wider spectrum of activity against different carbapenemase-producing bacteria, revealing a new strategy to combat β-lactamase-mediated antimicrobial resistance.

## Graphical abstract

**Figure:**
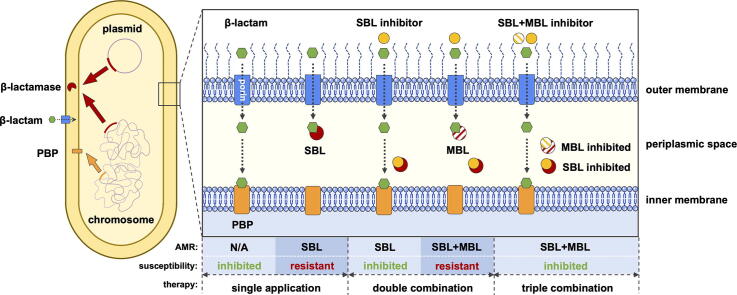

## 1 Introduction

β-Lactams, including penicillins, cephalosporins, and carbapenems, are among the most used anti-infective medicines in both hospital and community settings; they are also widely used in animal farming [1], [2]. Resistance to β-lactams occurs via multiple mechanisms, among which the most therapeutically relevant involves the production of β-lactamases, which catalyze β-lactam hydrolysis to yield inactive β-amino acids [1]. In mechanistic terms, β-lactamases are classified into nucleophilic serine-β-lactamases (SBLs; Ambler classes A, C, D) and zinc-ion-dependent metallo-β-lactamases (MBLs; Ambler class B, subclasses B1–B3) [3], [4]. Two main strategies have been employed to mitigate β-lactamase resistance: modification of the β-lactam antibiotic to avoid or inhibit β-lactamase catalysis, with monobactams and carbapenems being examples of this; or use of a β-lactamase inhibitor in combination with a β-lactam antibiotic. Examples of clinically important SBL inhibitors include clavulanic acid, tazobactam, and sulbactam, all of which are β-lactams, and the more recently developed avibactam (AVI), which does not contain a β-lactam ring [5] but still maintains the ability to react with SBLs in a covalent and reversible way [6], [7], [8]. AVI manifests a broad spectrum of activity against many classes A, C, and D SBLs but—like other clinically used SBL inhibitors—is ineffective in combating MBLs [6], [9]. Pioneering work on combatting β-lactamase activity focused on the SBLs because of their clinical relevance relative to MBLs; however, in recent times, B1-subfamily MBLs (e.g., New Delhi metallo-β-lactamase (NDM), Verona integron-encoded metallo-β-lactamase (VIM), and imipenemase (IMP) MBLs) have become more widespread and are endemic in some regions [3]. It is particularly worrying that MBLs are active against carbapenems, which are used frequently in hospitals when other β-lactams have failed due to resistance. Furthermore, the co-existence of SBL- and MBL-type carbapenemases in the same strains has been reported in 31 countries [10] (Fig. S1 and Table S1 in Appendix A).

The variations in the MBL active sites, coupled with the need to avoid MBL-like enzymes with vital roles in human biology, make it challenging to identify MBL inhibitors with sufficient breadth of activity and lack of toxicity; however, several promising compound series are in development [11], [12]. Recently, indole carboxylate derivatives have been shown to be potent MBL inhibitors with the ability to restore *in vivo* carbapenem (meropenem (MEM)) activity against MBL-producing bacteria [13]. The binding mode of the indole-2-carboxylates (InCs) mimics that of bicyclic β-lactams and/or subsequently formed intermediates [13]: interestingly, the InCs inhibit MBLs in part by stabilizing the hydrolytic di-zinc bridging water/hydroxide [13]. However, the indole carboxylates do not potently inhibit SBLs.

Resistance against antibacterial drug combinations involving AVI—most importantly, ceftazidime (CAZ)–AVI–has emerged. Aside from MBLs, which are not inhibited by AVI, CAZ–AVI resistance involves substitutions at key residues of SBLs, mutations or elevated expression of *Klebsiella pneumoniae* carbapenemases (KPCs), enhanced activity of efflux pumps, mutations to porins, and mutations in the transpeptidase targets of β-lactam antibiotics [14], [15], [16], [17], [18], [19], [20], [21], [22]. Hitherto, to the best of our knowledge, there are no reports available on the potential mechanisms of resistance against β-lactam plus indole carboxylate combinations.

Here, we report data on the triple combination of a β-lactam antibiotic, MEM, a broad spectrum SBL inhibitor (AVI), and a broad spectrum B1 MBL inhibitor (InC58), with the aim of maximizing the bactericidal spectrum of activity and investigating the frequency of resistance (FoR) and resistant mechanisms associated with the use of a potent MBL inhibitor (InC58).

## 2 Materials and methods

### 2.1 Antimicrobial compounds and bacterial strains

InC58 was synthesized at the Latvian Institute of Organic Synthesis, according to the reported procedure [13]. MEM, imipenem (IMI), taniborbactam (TAN), and AVI were obtained from Biosynth Carbosynth (Switzerland), Sigma Aldrich (USA), MedChemExpress (USA), and Biorbyt (USA), respectively. A MEM-resistant panel of 51 strains covering different classes of β-lactamase was obtained from the collections (Burden of Antibiotic Resistance in Neonates from Developing Societies (BARNARDS), India, Pakistan, Vietnam, Serbia) of the Ineos Oxford Institute for Antimicrobial Research and isolates that are the property of Marek Gniadkowski.

### 2.2 Susceptibility assay

Minimum inhibitory concentrations (MICs) were determined using the agar dilution method, according to the guidelines of the Clinical and Laboratory Standards Institute (CLSI, USA) [23]. Mueller Hinton Agar (Millipore, USA) was used in the assay. MEM, TAN, and AVI were dissolved in H_2_O, while IMI and InC58 were respectively dissolved in phosphate buffer and dimethyl sulfoxide (DMSO). For double and triple combinations, the concentrations of β-lactamase inhibitors were fixed at 4 or 2 mg·L^−1^, while the concentrations of MEM were varied. *Escherichia coli* (*E. coli*) ATCC 25922 and *Klebsiella pneumoniae* (*K. pneumonia*) ATCC 700603 were used as quality control strains. MIC_50_ is defined as the MIC value that inhibits the growth of higher or equal to 50% isolates [24].

### 2.3 FoR determination

FoR was tested against a bacterial panel (number of strains (*n*) = 19) susceptible to MEM–InC58 (InC58 at 4 mg·L^−1^), including *Klebsiella* spp., *E. coli*, *Enterobacter hormaechei* (*E. hormaechei*), *Serratia marcescens* (*S. marcescen*), and *Citrobacter sedlakii* (*C. sedlakii*). Overnight cultures of bacteria were suspended in saline solution to approximately 10^7^ colony-forming unit per milliliter (CFU·mL^−1^). A ten fold dilution series of the suspension was inoculated on nutrient media for CFU determination. A 0.1 mL aliquot of the suspension was spread onto nutrient media containing both MEM (4×MIC) and InC58 (4 mg·L^−1^). Numbers of colonies were counted after 24 or 48 h of incubation at 37 °C and were confirmed for their resistant phenotype via MIC determination. The FoR was determined by dividing the number of resistant colonies growing on drug-containing media by the total CFU in the initial inoculum.

### 2.4 Growth curve

To investigate the effect on bacterial growth and fitness by the development of spontaneous resistance, the parental and mutant strains from the FoR assay were inoculated at approximately 10^6^ CFU·mL^−1^ and cultivated at 37 °C in Luria–Bertani (LB) broth. For acquiring growth curves, a spectrophotometer (BMG Labtech; Thermo Fisher Scientific, USA) was used to test optical density at 600 nm wavelength (OD_600nm_) at an interval of every half hour.

### 2.5 Stability assay

The colony counting method was used to examine the stability of spontaneous mutants. Mutants were inoculated at approximately 10^6^ CFU·mL^−1^, grown at 37 °C (200 r·min^−1^), and passaged consecutively for 12 days by 1024 fold dilution in LB broth containing no antibiotics. Bacterial cultures were taken every four days, diluted and inoculated onto antibiotic-free and antibiotic-containing (1/8 and 1/2 MIC values of MEM–InC58) media to assess the retention ratio of the respective resistant phenotypes.

### 2.6 Whole genome sequencing

The QIAamp DNA mini kit (Qiagen, Germany) was used for bacterial DNA extraction, with an additional step of ribonuclease (RNase), using a Qiacube machine (Qiagen). Extracted DNA was quantified using a Qubit fluorometer (Invitrogen, USA). For long read sequencing, the extracted genomic deoxyribonucleic acid (gDNA) was purified and concentrated using Mag-Bind total pure NGS magnetic beads (Omega Bio-Tek, USA). Sequencing libraries were prepared using the Oxford Nanopore Technologies (UK) SQK-RBK110.96 rapid barcoding kit. Sequencing was carried out using a R9.4.1 flow cell on a MinION device (Oxford Nanopore Technologies) and base-calling was performed using the Guppy 6.1.2 toolkit integrated in the MINknow software. Short read sequencing was carried out as described previously [25], with minor modification. In brief, the Nextera XT V3 kit (Illumina, USA) and bead-based normalization were used to prepare genomic libraries. Whole genome sequencing (WGS) was carried out using V3 chemistry on the Illumina MiSeq platform for generating paired-end reads of up to 300 base pairs (bp).

### 2.7 Genetic analysis of bacterial strains

Long reads were assembled using Flye 2.9.1-b1780 [26] and polished using Medaka 1.7.3 (UK), followed by short reads polishing using Pilon 1.24 [27], Polypolish 0.5.0 [28], and Polca (MaSuRCA 4.1.0 [29]). The polished genomes were submitted to ResFinder 4.1 to identify β-lactamase genes [30], [31], [32]. KmerFinder 3.2 [33], [34], [35] and matrix-assisted laser desorption ionization time-of-flight mass spectrometry (MALDI-TOF MS; Bruker, USA) were used to identify bacterial species. For genetic variation analysis, the polished genomes were considered as reference genomes for the downstream analysis. Variations between the reference genomes and their corresponding mutants (short reads) were analyzed using Snippy 4.6.0†. Errors in the polished assembly of the reference genomes and resistance-unrelated variations were excluded by remapping the short read data of the parental strains to the reference genomes. Amino acid sequence alignments were performed using Geneious Prime 2023.2.1 (New Zealand).

### 2.8 Accession numbers

WGS data were deposited in the Sequence Read Archive (SRA) under the BioProject ID PRJNA984017.

## 3 Results

### 3.1 Efficacy of the triple combination

**Fig. 1:**
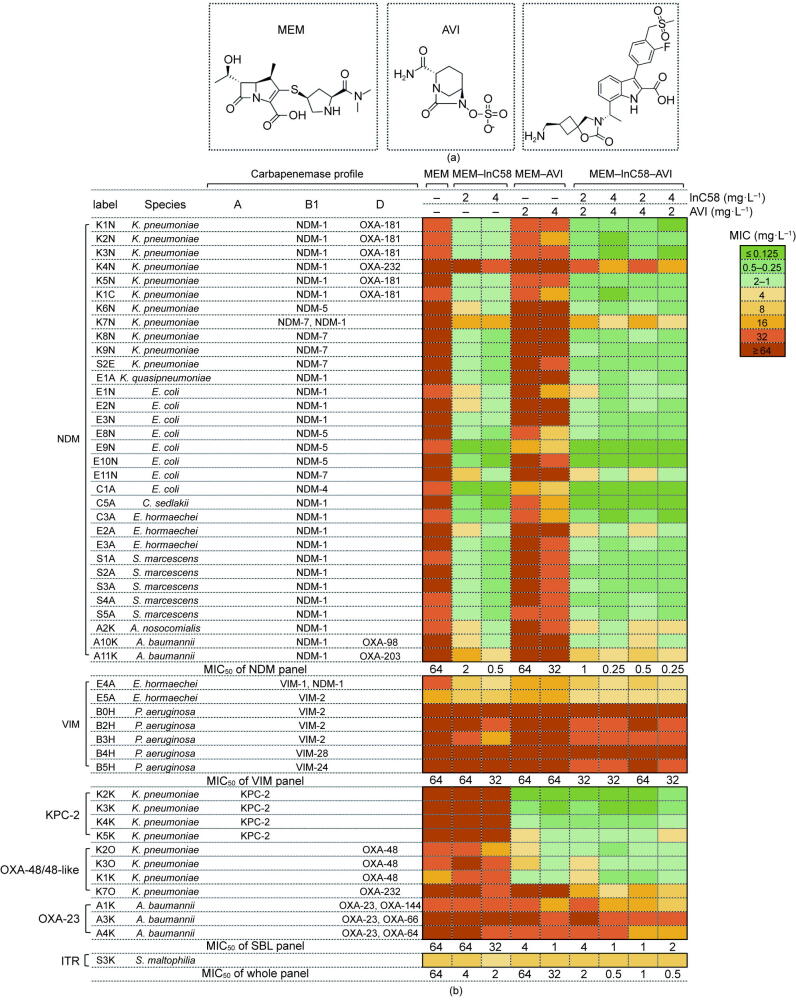
(a) The structures of MEM [36], AVI [6], and InC58 [13] were derived from reported studies. Reproduced from Ref. [6], [13], [36] with permission; (b) antibacterial activity of MEM alone, MEM in combination with AVI or InC58, and the MEM–InC58–AVI triple combination. TR: intrinsic resistance. Detailed information of the strain panel is listed in Table S2 in Appendix A. *K. quasipneumoniae: Klebsiella quasipneumoniae, A. nosocomialis: Acinetobacter nosocomialis*.

A panel (Table S2 in Appendix A) of 51 Gram-negative bacteria covering clinically relevant SBL and MBL carbapenemases was used to assess the efficacy of MEM in combination with InC58 and/or AVI (Fig. 1 [6], [13], [36]). The MEM–InC58 combination was active against different types of NDM-producing strains in the panel but was ineffective against VIM-positive isolates. When added at 4 mg·L^−1^, InC58 lowered the MIC_50_ of our NDM panel (*n* = 33) to 0.5 mg·L^−1^—a 128 fold decrease compared with MEM alone (MIC_50_ = 64 mg·L^−1^). The MIC of VIM-carrying *Enterobacter* spp. decreased 2 to 8 fold (MICs: 4–8 mg·L^−1^) after the addition of InC58 (4 or 2 mg·L^−1^) to MEM, while the VIM-carrying *P. aeruginosa* maintained high-level resistance to MEM–InC58 (MICs ≥ 16 mg·L^−1^).

The MEM–AVI combination was potent against strains with different SBL carbapenemases (oxacillinase (OXA)-48 and KPC-2) but was inactive against OXA-23-carrying *Acinetobacter baumannii*. Compared with MEM alone, the MICs of MEM–AVI (AVI at 4 mg·L^−1^) against OXA-48- or KPC-2-positive *K. pneumoniae* were more than or equal to 16 fold lower (OXA-48 MIC: 1–2 mg·L^−1^; KPC-2 MIC: 0.125–1 mg·L^−1^).

The results for MEM–InC58–AVI (Fig. 1) showed that the activities of InC58 and AVI were complementary, providing a wider spectrum of activity against the MBL and SBL combination. The presence of both InC58 and AVI at 4 mg·L^−1^ lowered the MEM MIC_50_ of all isolates in our panel to 0.5 mg·L^−1^—64 fold lower compared with the MIC_50_ (32 mg·L^−1^) of MEM–AVI (AVI at 4 mg·L^−1^) and four fold lower than the MIC_50_ (2 mg·L^−1^) of MEM–InC58 (InC58 at 4  mg·L^−1^).

Compared with the inhibition observed at 2 mg·L^−1^, InC58 and AVI both manifested slightly better inhibition at 4 mg·L^−1^ in both the double and triple combinations. In the double combination, the MIC_50_ of MEM–InC58 was reduced from 2 mg·L^−1^ (InC58 at 2 mg·L^−1^) to 0.5 mg·L^−1^ (InC58 4 mg·L^−1^) against the NDM panel (*n* = 33). The MIC_50_ of MEM–AVI was reduced from 4 mg·L^−1^ (AVI at 2 mg·L^−1^) to 1 mg·L^−1^ (AVI at 4 mg·L^−1^) against the SBL panel (*n* = 11). With the triple combination, a MEM concentration of 0.5 mg·L^−1^ inhibited 59% of the isolates of the whole panel when combined with AVI (4 mg·L^−1^) and InC58 (4 mg·L^−1^), while the same MEM concentration inhibited only 33%–51% of the strains when combined with AVI (2 mg·L^−1^)–InC58 (2 mg·L^−1^), AVI (2 mg·L^−1^)–InC58 (4 mg·L^−1^), or AVI (4 mg·L^−1^)–InC58 (2 mg·L^−1^).

### 3.2 Frequency of spontaneous resistance

**Fig. 2:**
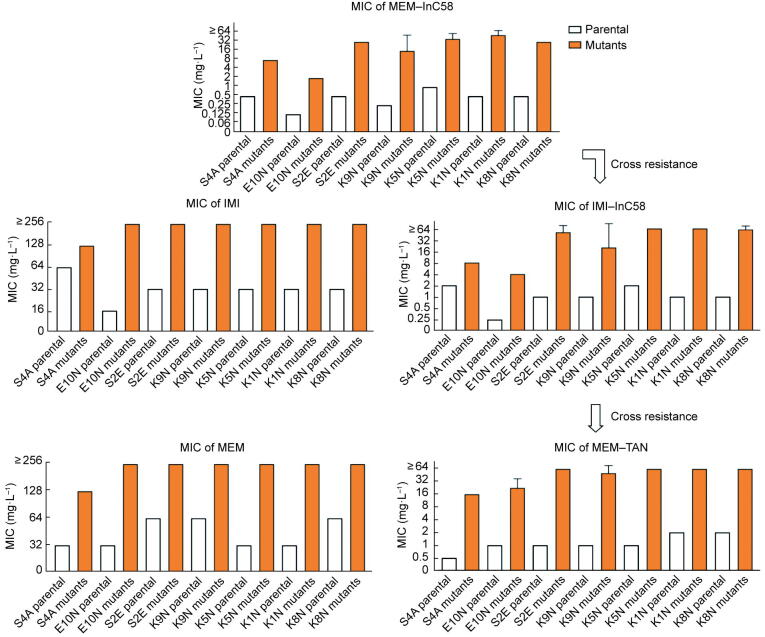
The MICs of mutants against different carbapenems (MEM and IMI) and double combinations (MEM–InC58, IMI–InC58, and MEM–TAN) with β-lactamase inhibitor concentration at 4 mg·L^−1^.

FoR was undertaken against strains that showed susceptibility to the MEM–InC58 combination (InC58 at 4 mg·L^−1^). Spontaneous mutants derived from *K. pneumoniae* isolates ranged from 2.88 × 10^−7^ to 1.13 × 10^−6^. Two *E. coli* mutants and two *S. marcescens* mutants were identified with FoR rates of 2.22 × 10^−7^ and 4.00 × 10^−7^, respectively (Table 1). Susceptibility testing (Fig. 2) showed that most of the *K. pneumoniae* mutants (24/25) were highly resistant to MEM–InC58 (InC58 at 4 mg·L^−1^), with MICs higher than or equal to 32 mg·L^−1^. The MICs of MEM–InC58 (InC58 at 4 mg·L^−1^) for both the *E. coli* and *S. marcescens* mutants increased by 16 fold, to 2 and 8 mg·L^−1^, respectively.

**Table 1:** FoR against MEM–InC58 (InC58 at 4 mg·L^−1^).

Strains	Species	Carbapenemase profile	MIC of MEM–InC58 (mg·L^−1^)	FoR (4× MIC)
K1N	*K. pneumoniae*	NDM-1, OXA-181	1	7.69 × 10^−7 a^
K5N	*K. pneumoniae*	NDM-1, OXA-181	1	5.08 × 10^−7 a^
K8N	*K. pneumoniae*	NDM-7	0.25	1.13 × 10^−6 a^
K9N	*K. pneumoniae*	NDM-7	0.25	2.88 × 10^−7 a^
S2E	*K. pneumoniae*	NDM-7	0.25	4.92 × 10^−7 a^
E10N	*E. coli*	NDM-5	0.125	2.22 × 10^−7 b^
S4A	*S. marcescens*	NDM-1	0.5	4.00 × 10^−7 a^

^a^ Mutants appeared after 24 h incubation; ^b^ Mutants appeared after 48 h incubation.

Compared with the parental strains, the MEM MICs of all mutants were elevated by larger than or equal to four fold, showing enhanced resistance to MEM (Fig. 2). In addition, when the concentration of InC58 was increased from 4 to 16 mg·L^−1^, the MEM–InC58 MICs of approximately two-thirds of the mutants (20/29) were the same or were within a two fold variation (Fig. S2 in Appendix A).

### 3.3 Cross resistance of spontaneous mutants

To investigate whether the spontaneous resistance against MEM–InC58 was due to an increase in resistance specifically to MEM and/or InC58, cross-resistance assays were carried out (Fig. 2). Interestingly, when InC58 in the double combination was replaced by TAN (another β-lactamase inhibitor with activity against MBLs and SBLs), the double combination still failed to effectively inhibit the growth of all the mutants, with MICs higher than or equal to 32 mg·L^−1^ for *K. pneumoniae* mutants and higher than or equal to 16 mg·L^−1^ for *E. coli* and *S. marcescens* mutants being observed. When MEM was replaced with IMI in a double combination with InC58, all the mutants presented resistance to IMI–InC58. Most *K. pneumoniae* mutants (24/25) showed IMI–InC58 MICs of higher than or equal to 32 mg·L^−1^, and a larger than or equal to four fold increase in MICs was observed for the remaining *K. pneumoniae*, *E. coli*, and *S. marcescens* mutants compared with their parental strains. These results indicated that the resistant mutants employed mechanisms against the different carbapenem and β-lactamase inhibitor combinations.

### 3.4 Fitness cost of spontaneous mutants

**Fig. 3:**
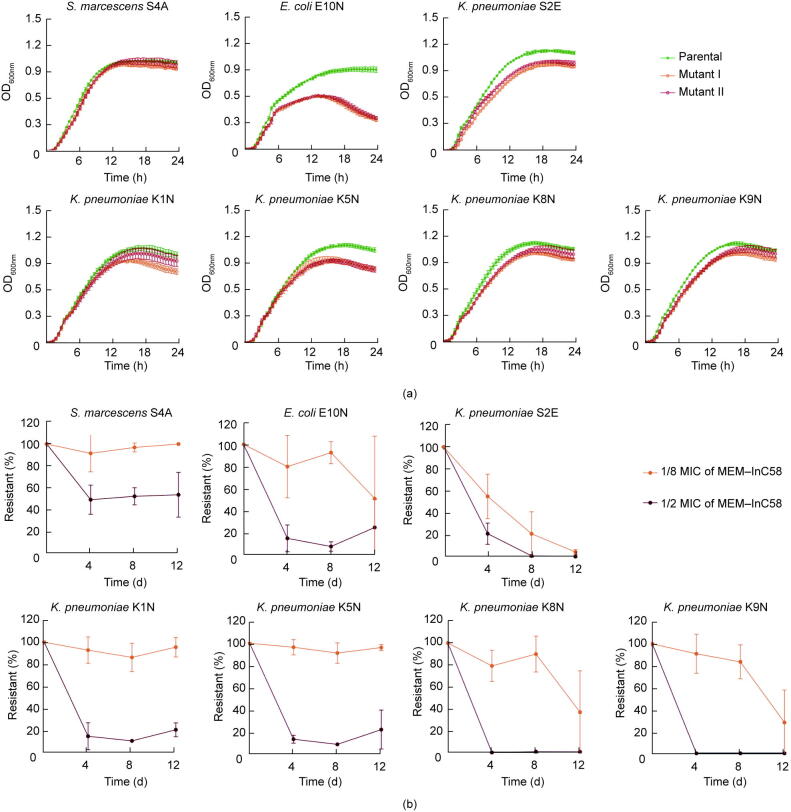
Fitness and stability of spontaneous resistant mutants to MEM–InC58. (a) Growth curves of mutants and corresponding parental strains; (b) stability of resistant phenotypes among spontaneous mutants during passages in the absence of antibiotics.

To assess whether spontaneous resistance to MEM–InC58 affects bacterial growth and fitness, we compared growth curves between parental strains and two of the mutants (Fig. 3(a)). The *E. coli* mutants showed the most substantial suppression of growth, with a decrease of approximately 0.5 OD_600nm_ at 24 h, compared with the parental strain. The *S. marcescens* mutants presented a relatively lower fitness cost, with a slight delay during the log phase. Of the *K. pneumoniae* mutants, 9/10 demonstrated distinguishable growth retardation except one, K1N-M2 (resistant mutant 2 of K1N), for which only a small reduction in growth was observed in comparison with the parental strain (Fig. 3(a)).

### 3.5 Stability of spontaneous mutants

On media containing 1/2 MIC of MEM–InC58 (InC58 at 4 mg·L^−1^), the resistant phenotype of most mutants was unsustainable (Fig. 3(b)). We observed a sharp decline (≥ 79%) in the average retention ratio of the resistant phenotype on day four for most (6/7) of the mutants. The mutant of *S. marcescens* S4A manifested relatively higher stability compared with the other mutants, with the average retention ratio being maintained at approximately 50% from days 4 to 12. On media containing 1/8 MIC of MEM–InC58 (InC58 at 4 mg·L^−1^) (Fig. 3(b)), the mutants from *S. marcescens* S4A and *K. pneumoniae* K1N and K5N maintained their resistant phenotype, with an average retention ratio of higher or equal to 86% during days 4–12. The retention ratio of other mutants showed a decreasing tendency during the 12 day passage in the absence of antibiotics. These results suggested that, without the selective pressure of antibiotics, most mutants were incapable of maintaining resistance to the combination of MEM–InC58. After passaging without antibiotic pressure, we acquired new mutants that exhibited restored sensitivity against MEM–InC58, from resistant mutants of *E. coli* E10N and *K. pneumoniae* S2E, K8N, and K9N (Table S3 in Appendix A).

### 3.6 Genetic variation analysis of spontaneous resistance

**Fig. 4:**
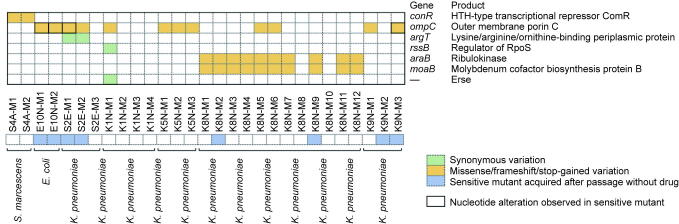
Genetic variations of spontaneous resistant mutants to MEM–InC58. HTH: helix–turn–helix; RpoS: RNA polymerase, sigma S subunit.

To investigate the mechanism of resistance against MEM–InC58, the resistant mutants generated in the FoR assay and their corresponding susceptible mutants were sequenced and compared with the wildtype reference strains to determine differences (Fig. 4; Table S4 in Appendix A). Among the resistant mutants, all the *E. coli* mutants (*n* = 2) and 40% of the *K. pneumoniae* mutants (10/25) presented mutations in the *ompC*, which encodes outer membrane porin C (OmpC, where the OmpC family includes the OmpK36 that is well-known in *K. pneumoniae* [37]). Interestingly, both the *E. coli* resistant mutants (*n* = 2) possessed the Arginine295Proline (Arg295Pro) OmpC variant compared with the wildtype reference strain, while their corresponding susceptible mutants (*n* = 4) all presented the Arginine295Serine (Arg295Ser) substitution at the same position (Table S4 and Fig. S3 in Appendix A). Of the resistant mutants of *K. pneumoniae* that had *ompC* mutations (*n* = 10), two gained a stop code in the middle of *ompC*; three suffered frameshifts due to the insertion of base pairs; and the remaining five presented missense mutations that changed a single amino acid of OmpC. We acquired sensitive mutants (*n* = 5) from the mutants that gained a stop code (S2E-M, *n* = 2, Gln124*) and a frameshifted mutant (K9N-M3). However, all of them maintained the same *ompC* stop codon and frameshift mutations, with the only exception being the conversion of Gln124* into Glutamine124Leucine (Gln124Leu; missense mutation).

Both the resistant mutants of *S. marcescens* had the same frameshift on the *comR* gene, whose disruption (a transposon insertion) has been reported to reduce copper permeability across the bacterial outer membrane [38].

In addition, the majority (10/12) of mutants from K8N possessed altered *araB* and *moaB* genes that encode ribulokinase and molybdenum cofactor biosynthesis protein B, respectively. The corresponding susceptible mutants presented the same mutations in the *araB* and *moaB*. At present, we have not found any direct relationship between the above two genes and antibiotic resistance.

## 4 Discussion

The increasing global prevalence of SBL and MBL carbapenemases is a threat to the broad efficacy of β-lactams in combination with a single β-lactamase inhibitor [3]. Previous studies have investigated the utility of certain triple combinations against carbapenem-resistant Gram-negative bacteria, such as two monobactams with clavulanic acid [39], β-lactams combined with AVI and a tobramycin-cyclam conjugate [40], β-lactams combined with two SBL inhibitors (sulbactam and AVI) [41], or imipenem combined with relebactam (SBL inhibitor) and cilastatin (a renal dehydropeptidase inhibitor) [42]. To the best of our knowledge, our work is the first study that examines the use of both MBL and SBL-selective inhibitors in combination with a carbapenem, aimed at achieving optimized coverage against various carbapenemase-producing bacteria.

Our results reveal the clinical potential of a triple combination to achieve extended broad-spectrum antibacterial activity against multiple and diverse SBL- and MBL-bearing Gram-negative strains. The two β-lactamase inhibitors in our triple combination act in a complementary way: AVI mitigates the acylation by SBLs [6], [8] and InC58 negates the hydrolysis of MBLs [13]. The results illustrate how a combination of SBL and MBL inhibitors can extend the efficacy of a carbapenem such as MEM and, likely, other β-lactam antibiotics.

In line with the pIC50s (pIC50 = −log(IC_50_); IC_50_: half-maximal inhibitory concentration) of AVI and InC58 against different β-lactamases (Table S5 in Appendix A) [13], the double combinations of MEM–AVI and MEM–InC58 are respectively active against SBL- and MBL-type carbapenemases, with the exception of OXA-23-positive *A. baumannii* and VIM-positive *P. aeruginosa*. MEM–AVI inhibited OXA-48 and KPC-2 producers in the panel, in accordance with the results of an earlier study [9], although the combination was ineffective against the OXA-23-positive *A. baumannii*. The MEM–InC58 combination demonstrated good activity against NDM-positive isolates, consistent with previous research [13], but was less effective versus VIM-positive *Enterobacter* spp. and even less so for VIM-producing *P. aeruginosa*. These exceptions may relate to the poor permeability of antimicrobial compounds, as previously described [43], [44], [45]. Brem et al. [13] also reported the limited activity of InC49, an analogue of InC58, against VIM-producing *P. aeruginosa* and NDM-producing *A. baumannii*, when combined with MEM or imipenem. Similarly, the levels of resistance against CAZ–AVI for *P. aeruginosa* and *A. baumannii* were higher than those for Enterobacteriaceae [15]. Another reason is that InC58 and AVI are not potent to VIM and OXA-23, respectively. This was evidenced by previous work showing that the pIC50 of InC58 to VIMs was lower than that to NDMs [13]. AVI was also reported to be inefficient toward OXA-23 [46]. Accordingly, the use of MEM–InC58–AVI should be avoided when treating infections caused by VIM-carrying *P. aeruginosa* and OXA-23-carrying *A. baumannii*; however, this limitation might be overcome by the use of an alternative antibacterial drug.

The application of new antibiotics is very likely to be accompanied by the emergence of bacterial resistance due to selective pressure. Earlier studies have investigated resistance mechanisms (apart from the expression of MBLs) against CAZ–AVI, which were found to be associated with mutations in critical sites (e.g., the Ω-loop) of different SBLs, elevated activity of efflux pumps, mutations in PBPs (penicillin-binding proteins) or porins, and enhanced expression of KPC [14], [15], [16], [17], [18], [19], [20], [21], [22]. However, resistance against the novel MBL inhibitors, such as InC58, has not been investigated in detail.

In our FoR assays, spontaneous resistant mutants were generated against the MEM–InC58 combination. All the tested mutants manifested a retarded growth rate compared with their parental strains, indicating a fitness cost brought by the acquisition of resistance to MEM–InC58. The results of stability assays suggested that, without the selective pressure of antibiotics, the mutants did not maintain the original level of resistance to MEM–InC58, although the stability of the resistant phenotype of the *S. marcescens* mutant was relatively higher than those of the mutants from other species in our study. Moreover, these mutants not only became less sensitive to MEM–InC58 or to MEM itself but also generated cross resistance to IMI-InC58 and MEM–TAN, suggesting that the resistance mechanism non-specifically targets different carbapenems and β-lactamase inhibitors.

The analysis of the WGS data indicated three potential mechanisms of resistance to MEM–InC58. Notably, among the resistant mutants to MEM–InC58, no amino acid residue substitutions in the β-lactamases were identified. From the perspective of compound structures, InC58 does not contain a reactive carbonyl group; hence, β-lactamase-mediated resistance to it seems unlikely. In contrast, while AVI does not contain a β-lactam, its mechanism of action—that is, the reaction of its cyclic urea with the nucleophilic serine of the SBLs—suggests that SBL-mediated resistance to it may emerge. For example, resistance related to mutations on the Ω-loop in KPCs have been reported after exposure to CAZ–AVI [14], [20], [21].

For the two *S. marcescens* mutants, the sequence analysis showed that both had a frameshift in the *comR*. ComR is a repressor that regulates the expression of ComC, where the latter is a protein on the outer membrane that lowers the permeability of the outer membrane to copper [38]. Therefore, disruption of *comR* could indirectly decrease the copper permeability into bacteria [38]. As copper ions inhibit the activity of NDM-1 [47], it is possible that the disruption of *comR* may improve NDM-1 activity in *S. marcescens* mutants. However, given that InC58 binds to the active-site zinc ions of MBLs, other roles for *comR* and metal ions in resistance to InC58 are also possible.

All of the *E. coli* mutants and 40% of the *K. pneumoniae* mutants had mutations in the *ompC* gene, which encodes OmpC. In earlier work, reduced expression of *ompC* was observed in bacteria under carbapenem stress, supporting a relationship between *ompC* and carbapenem resistance [48]. Porins are protein channels on the bacterial outer membrane; they are involved in the entry of multiple compounds, including antibiotics [37]. Mutations to porins, including *ompC*, have been linked to limited uptake of β-lactams [37], [49] and have potential to hinder the entry of β-lactamase inhibitors into bacteria. After the passaging of resistant mutants without antibiotic pressure, we acquired sensitive mutants of *E. coli* and *K. pneumoniae* that restored susceptibility to MEM–InC58. All the sensitive mutants of *E. coli* (*n* = 4) had the Arg295Ser mutation on OmpC, making them distinguishable from the resistant ones (*n* = 2, Arg295Pro); this finding suggests that Arg295 may be an important site with respect to resistance against MEM–InC58. However, for susceptible mutants of *K. pneumoniae*, compared with their resistant counterparts, most (4/5) kept the same stop code and frameshift mutations on *ompC*. This phenomenon suggests that the disruption of porin C is not the only factor promoting insusceptibility to MEM–InC58 among these resistant mutants of *K. pneumoniae*. In addition to mutations on the nucleotide level, other resistance mechanisms likely exist.

The extent to which resistance emerges in a clinical context to single drugs versus double and triple drug combinations will be interesting to monitor. However, the knowledge that some β-lactam plus SBL inhibitor combinations are still vital medicines, such as Augmentin (amoxycillin and clavulanic acid), suggests that new combination therapies have substantial clinical potential.

## 5 Conclusions

The triple combination of MEM with InC58 (a novel MBL inhibitor) and AVI (an SBL inhibitor) showed a much broader spectrum of antimicrobial activity against different β-lactamase-producing bacteria when compared with dual combinations. These results reveal a new strategy for combating β-lactamase-mediated antimicrobial resistance.

## Footnotes

† https://github.com/tseemann/snippy.
