# Supplementary material for: The Triple Combination of Meropenem, Avibactam, and a Metallo-β-Lactamase Inhibitor Optimizes Antibacterial Coverage Against Different β-Lactamase Producers
Source: Engineering (Beijing). 2024 Jul;38:124–32. doi: 10.1016/j.eng.2024.02.010 (PMC11913740; doi:10.1016/j.eng.2024.02.010)
Supplement: Supplementary Data 1 [file mmc1.docx]

**Supplementary Materials**

**The triple combination of** **meropenem, avibactam and a metallo β-lactamase inhibitor** **optimizes antibacterial coverage against different** **β-lactamase producers**

Zhuoren Ling^a*^, Alistair James Macdonald Farley^b^, Aditya Lankapalli^a^, Yanfang Zhang^a^, Shonnette Premchand-Branker^a^, Kate Cook^a^, Andrei Baran^c^, Charlotte Gray-Hammerton^a^, Claudia Orbegozo Rubio^a^, Edgars Suna^c^, Jordan Mathias^d^, Jürgen Brem^b,e^, Kirsty Sands^a^, Maria Nieto-Rosado^a^, Maria Mykolaivna Trush^a^, Nadira Naznin Rakhi^a^, Willames Martins^a^, Yuqing Zhou^a^, Christopher Joseph Schofield^b^, Timothy Walsh^a^

^a^Department of Biology and the Ineos Oxford Institute for Antimicrobial Research, University of Oxford, Oxford OX1 3RE, UK

^b^Department of Chemistry, Chemistry Research Laboratory and the Ineos Oxford Institute for Antimicrobial Research, University of Oxford, Oxford OX1 3TA, UK

^c^Latvian Institute of Organic Synthesis, Riga LV-1006, Latvia

^d^Department of Medical Microbiology, Division of Infection and Immunity, School of Medicine, Cardiff University, Cardiff CF14 4XN, UK

^e^Enzymology and Applied Biocatalysis Research Center, Faculty of Chemistry and Chemical Engineering, Babes-Bolyai University, Cluj-Napoca, RO-400028, Romania

*** Corresponding author:** Zhuoren Ling, E-mail: lingacademic@163.com

**Table S1.** Global distribution of bacteria co-harbouring SBL- (KPCs, OXA-23 and OXA-48/48-like) and MBL- (NDMs, VIMs, IMPs) carbapenemase genes.

| Country | Genotype | Species | Number of  Isolates | Year of  Isolation | Source | Citation |
| --- | --- | --- | --- | --- | --- | --- |
| China | NDM-1+KPC-2 | *R. ornithinolytica* | 1 | - | environment | [1] |
|  | NDM-1+KPC-2 | *K. pneumoniae* | 1 | 2017 | human | [2] |
|  | NDM-1+KPC-2 | *C. freundii* | 1 | 2013 | human | [3] |
|  | NDM-5+KPC-2 | *E. coli* | 1 | 2018 | human | [4] |
|  | NDM-1+KPC-2 | *R. planticola* | 1 | - | human | [5] |
|  | NDM-1+KPC-2+IMP-4 | *K. michiganensis* | 1 | 2021 | human | [6] |
|  | NDM-1+KPC-2 | *E. cloacae* | 1 | 2014 | human | [7] |
|  | NDM-1+KPC-2 | *K. pneumoniae* | 1 | 2017 | human | [8] |
|  | NDM-1+KPC-2 | *K. pneumoniae* | 1 | 2013-2014 | human | [9] |
|  | NDM-1+KPC-2 | *C. freundii* | 1 | 2015 | environment | [10] |
|  | NDM-1+IMP-4+KPC-2 | *C. freundii* | 1 | 2013 | human | [11] |
|  | KPC-2 +VIM-1 | *K. pneumoniae* | 1 | 2008 | human | [12] |
|  | KPC-2+IMP-4 | *K. pneumoniae* | 3 | 2008-2009 | human | [13] |
|  | IMP-4+KPC-2 | *R. ornithinolytica* | 1 | 2014 | human | [14] |
|  | KPC-2+IMP-4 | *K. pneumoniae* | 1 | 2011-2012 | human |  |
|  | KPC-2+IMP-4 | *E. cloacae* | 1 | 2011-2012 | human |  |
|  | KPC-2+IMP-4 | *K. oxytoca* | 1 | 2011-2012 | human |  |
|  | KPC-2+IMP-26 | *K. pneumoniae* | 3 | 2011-2012 | human |  |
|  | KPC-2+IMP-26 | *E. cloacae* | 2 | 2011-2012 | human |  |
|  | KPC-2+IMP-8 | *C. freundii* | 1 | 2011-2012 | human |  |
|  | KPC-2+NDM-1 | *K. oxytoca* | 1 | 2011-2012 | human | [15] |
|  | KPC-2+IMP-4 | *K. pneumoniae* | 1 | 2010 | human | [16] |
|  | KPC-2+IMP-30 | *E. coli* | 1 | 2015 | human | [17] |
|  | OXA-48+NDM-1 | *K. pneumoniae* | 1 | 2015 | human | [18] |
|  | OXA-181+NDM-4 | *K. pneumoniae* | 3 | 2019 | human | [19] |
|  | IMP+OXA-23 | *Acinetobacter* spp. | 90 | 2012-2013 | human | [20] |
|  | IMP-38+OXA-48+NDM-1 | *K. pneumoniae* | 2 | 2017; 2016 | human | [21] |
|  | OXA-48+NDM-1 | *K. pneumoniae* | 1 | 2016 | human |  |
| Brazil | KPC-2+NDM-1 | *E. cloacae* | 1 | 2013-2015 | human | [22] |
|  | NDM+VIM+KPC | *K. pneumoniae* | 5 | 2016-2017 | human | [23] |
|  | NDM+VIM+KPC+OXA-48 | *K. pneumoniae* | 3 | 2016-2017 | human |  |
| Egypt | NDM+OXA-23 | *A. baumannii* | 6 | 2017-2018 | human | [24] |
|  | KPC+NDM+OXA-23 | *A. baumannii* | 9 | 2017-2018 | human |  |
|  | KPC+NDM | *A. baumannii* | 4 | 2017-2018 | human |  |
|  | IMP+OXA-23 | *A. baumannii* | 1 | 2017-2018 | human |  |
|  | KPC+NDM+IMP | *A. baumannii* | 1 | 2017-2018 | human |  |
|  | NDM+OXA-23+OXA-48+OXA-181 | *K. pneumoniae* | 1 | - | human | [25] |
|  | OXA-48+NDM | *K. pneumoniae* | 1 | - | human |  |
|  | OXA-48+OXA-181+NDM | *K. pneumoniae* | 1 | - | human |  |
|  | NDM-1+OXA-48 | Enterobacteriaceae | 9 | 2019 | human | [26] |
|  | OXA-23+NDM | *A. baumannii* | 14 | 2019-2021 | human | [27] |
|  | NDM-1 + OXA-48-like | *K. pneumoniae* | 2 | 2014 | human | [28] |
|  | VIM-1-like + OXA-48- like | *K. pneumoniae* | 3 | 2014 | human |  |
|  | NDM-1 + VIM-1-like + OXA-48-like | *K. pneumoniae* | 1 | 2014 | human |  |
|  | VIM-1-like + OXA-48- like | *E. coli* | 1 | 2014 | human |  |
|  | VIM-2 + OXA-48- like | *P. aeruginosa* | 2 | 2014 | human |  |
|  | OXA-48+IMP-1 | *K. pneumoniae* | 1 | 2015 | human | [29] |
|  | NDM-1+OXA-48 | *K. pneumoniae* | 15 | 2015 | human |  |
|  | OXA-48+NDM+VIM+IMP | *K. pneumoniae* | 4 | 2014-2016 | human | [30] |
|  | OXA-48+NDM+VIM | *K. pneumoniae* | 5 | 2014-2016 | human |  |
|  | OXA-48+VIM | *K. pneumoniae* | 7 | 2014-2016 | human |  |
|  | KPC+NDM+VIM+IMP | *K. pneumoniae* | 2 | 2014-2016 | human |  |
|  | OXA-48+NDM+VIM | *K. pneumoniae* | 1 | 2014-2016 | human |  |
|  | KPC+OXA-48+NDM+VIM+IMP | *K. pneumoniae* | 2 | 2014-2016 | human |  |
|  | OXA-48+NDM+VIM+IMP | *K. pneumoniae* | 3 | 2014-2016 | human |  |
|  | KPC+OXA-48+  NDM+VIM+IMP | *K. pneumoniae* | 1 | 2014-2016 | human |  |
|  | KPC+OXA-48+NDM+VIM | *K. pneumoniae* | 1 | 2014-2016 | human |  |
|  | NDM+IMP+OXA-48 | *K. pneumoniae* | 1 | 2014-2016 | human |  |
|  | NDM+OXA-48 | *K. pneumoniae* | 12 | 2015-2016 | human | [31] |
|  | NDM+VIM+KPC | *K. pneumoniae* | 8 | 2018-2019 | human | [32] |
|  | NDM+KPC | *K. pneumoniae* | 6 | 2018-2019 | human |  |
|  | NDM+KPC | *E. coli* | 1 | 2018-2019 | human |  |
|  | NDM+OXA-48 | *K. pneumoniae* | 4 | 2018-2019 | human |  |
|  | NDM+VIM+OXA-48 | *K. pneumoniae* | 4 | 2018-2019 | human |  |
|  | VIM+OXA-48 | *K. pneumoniae* | 2 | 2018-2019 | human |  |
|  | VIM+OXA-48 | *E. cloacae* | 1 | 2018-2019 | human |  |
|  | NDM+KPC+IMP | *E. coli* | 2 | 2018-2019 | human |  |
|  | NDM+VIM+KPC+IMP | *E. coli* | 1 | 2018-2019 | human |  |
|  | NDM+VIM+KPC+OXA-48 | *K. pneumoniae* | 1 | 2018-2019 | human |  |
|  | VIM+KPC+OXA-23 | *A. baumannii* | 7 | 2015-2016 | human | [33] |
|  | IMP+KPC+OXA-23 | *A. baumannii* | 3 | 2015-2016 | human |  |
|  | VIM+OXA-23 | *A. baumannii* | 2 | 2015-2016 | human |  |
|  | NDM+KPC+OXA-23 | *A. baumannii* | 4 | 2015-2016 | human |  |
|  | NDM+OXA-23 | *A. baumannii* | 4 | 2015-2016 | human |  |
|  | VIM+IMP+KPC+OXA-23 | *A. baumannii* | 1 | 2015-2016 | human |  |
|  | IMP+OXA-23 | *A. baumannii* | 1 | 2015-2016 | human |  |
| Venezuela | NDM-1+OXA-23 | *A. baumannii* | 2 | 2016 | human | [34] |
| Pakistan | KPC+VIM | *P. mirabilis* | 1 | 2017-2018 | human | [35] |
|  | OXA48+KPC+VIM | *E. cloacae* | 1 | 2017-2018 | human |  |
|  | NDM-1+KPC-2 | *K. pneumoniae* | 2 | 2013 | human | [36] |
|  | NDM-1+OXA-48 | *K. pneumoniae* | 7 | 2018-2019 | human | [37] |
|  | OXA-23+VIM | *A. calcoaceticus-baumannii* | 64 | 2020-2021 | human | [38] |
|  | OXA-23+NDM | *A. calcoaceticus-baumannii* | 21 | 2020-2021 | human |  |
|  | OXA-23+VIM+NDM | *A. calcoaceticus-baumannii* | 4 | 2020-2021 | human |  |
|  | NDM-1+OXA-181 | *K. pneumoniae* | 20 | 2015-2017 | human | [39] |
| India | OXA-181+NDM-5 | *K. pneumoniae* | 3 | 2013-2016 | human | [40] |
|  | OXA-48+NDM-1 | *E. coli* | 2 | - | human | [41] |
|  | OXA-48+VIM | *K. pneumoniae* | 10 | 2016-2017 | human | [42] |
|  | OXA-48+NDM | *K. pneumoniae* | 5 | 2016-2017 | human |  |
|  | OXA-48+IMP+NDM | *K. pneumoniae* | 1 | 2016-2017 | human |  |
|  | OXA-48+VIM+NDM | *K. pneumoniae* | 2 | 2016-2017 | human |  |
|  | NDM-1+OXA-23 | *A. baumannii* | 3 | 2010 | human | [43] |
|  | NDM+VIM+OXA-48 | *Klebsiella* spp. | 4 | 2018-2021 | human | [44] |
|  | NDM+VIM+OXA-48 | *Pseudomonas* spp. | 2 | 2018-2021 | human |  |
|  | NDM+VIM+OXA-48 | *Acinetobacter* spp. | 3 | 2018-2021 | human |  |
|  | NDM+OXA-48 | *Klebsiella* spp. | 1 | 2018-2021 | human |  |
|  | NDM+OXA-48 | *Pseudomonas* spp. | 2 | 2018-2021 | human |  |
|  | NDM-5+OXA-48 | *E. coli* | 3 | 2014-2016 | human | [45] |
|  | NDM-5+OXA-48 | *K. pneumoniae* | 2 | 2014-2016 | human |  |
|  | NDM-1+OXA-181 | *K. pneumoniae* | 1 | 2015-2017 | human | [39] |
| Bangladesh | NDM-5+OXA-48 | *P. aerugionsa* | 1 | 2016 | human | [46] |
|  | VIM-2+OXA-48 | *P. aerugionsa* | 3 | 2016 | human |  |
|  | VIM-5+OXA-48 | *P. aerugionsa* | 1 | 2016 | human |  |
|  | NDM+OXA-48 | *P. aerugionsa* | 1 | 2016 | human |  |
|  | NDM+OXA-48 | *P. hibiscicola* | 1 | 2016 | human |  |
|  | NDM+OXA-48 | *A. baumannii* | 1 | 2016 | human |  |
|  | NDM-1+OXA-48 | *A. baumannii* | 1 | 2016 | human |  |
|  | NDM-1+OXA-48 | *A. baumannii* | 1 | 2016 | human |  |
|  | NDM-1+OXA-48 | *P. stuartii* | 1 | 2016 | human |  |
|  | NDM-1+OXA-48 | *K. pneumoniae* | 1 | 2016 | human |  |
|  | NDM-5+OXA-48 | *K. pneumoniae* | 4 | 2016 | human |  |
|  | NDM+OXA-48 | *K. pneumoniae* | 3 | 2016 | human |  |
|  | OXA-23+NDM-1 | *A. baumannii* | 1 | 2016 | human | [47] |
|  | NDM-1+OXA-232 | *K. pneumoniae* | 2 | 2015-2017 | human | [39] |
| Turkey | OXA-48 + NDM | *K. pneumoniae* | 8 | 2012-2014 | human | [48] |
|  | NDM-1+OXA-48 | *K. pneumoniae* | 2 | 2013 | human | [49] |
|  | KPC+IMP+NDM | *P. aeruginosa* | 1 | 2015-2016 | human | [50] |
|  | KPC+NDM | *P. aeruginosa* | 6 | 2015-2016 | human |  |
|  | OXA-48+IMP+NDM | *P. aeruginosa* | 1 | 2015-2016 | human |  |
|  | OXA-48+NDM | *P. aeruginosa* | 6 | 2015-2016 | human |  |
|  | OXA-48+VIM | Enterobacteriaceae | 7 | 2010-2014 | human | [51] |
|  | OXA-48+NDM-1 | *P. aeruginosa* | 9 | 2017-2018 | human | [52] |
| Morocco | OXA-48+NDM | *K. pneumoniae* | 4 | 2019-2020 | human | [53] |
|  | OXA-48+NDM | *R. terrigena* | 9 | 2019-2020 | human |  |
|  | NDM-1+OXA-23 | *A. baumannii* | 27 | 2015 | human | [54] |
|  | OXA-23+NDM | *A. baumannii* and/or  *P. aeruginosa* | 4 | 2018-2021 | human | [55] |
| Spain | VIM-1+KPC-2 | *C. freundii* | 1 | 2014-2016 | human | [56] |
| Sri Lanka | NDM-4+OXA-181 | *E. hormaechei* | 1 | 2015 | human | [57] |
| Algeria | OXA-23+NDM-1 | *A. baumannii* | 2 | 2010-2013 | human | [58] |
|  | NDM-1+OXA-58 | *A. baumannii* | 1 | 2010-2013 | human |  |
| Tunisia | NDM-1+ OXA-48 | *K. pneumoniae* | 1 | 2016-2017 | human | [59] |
| Saudi Arabia | OXA-23+VIM | *A. baumannii* | 45 | 2008-2012 | human | [60] |
|  | OXA-48+NDM-1 | *K. pneumoniae* | 1 | 2012 | human | [61] |
|  | OXA-23 + NDM-1 | *A. baumannii* | 1 | 2012 | human |  |
|  | OXA-23+IMP | *A. baumannii* | 34 | 2015-2016 | human | [62] |
|  | OXA-23+IMP+VIM | *A. baumannii* | 21 | 2015-2016 | human |  |
|  | OXA-23+NDM+IMP+VIM | *A. baumannii* | 1 | 2015-2016 | human |  |
| Greece | KPC-2+VIM-1 | *K. pneumoniae* | 21 | 2007-2013 | human | [63] |
| Tanzania | IMP+KPC+OXA-48 | Gram-negative bacteria | 5 | 2018-2019 | clinical, colonization,  and hospital  environmental | [64] |
|  | IMP+KPC | Gram-negative bacteria | 4 | 2018-2019 |  |  |
|  | IMP+OXA-48 | Gram-negative bacteria | 1 | 2018-2019 |  |  |
| South Africa | OXA-23+IMP-1 | *A. baumannii* | 1 | 2016-2017 | human | [65] |
|  | OXA-23+IMP-1 | *A. baumannii* | 2 | 2016-2017 | human |  |
|  | NDM-OXA-48 | *A. baumannii* complex | 2 | - | human | [66] |
|  | NDM-OXA-48 | *K. pneumoniae* | 7 | - | human |  |
|  | IMP-VIM-OXA | *K. pneumoniae* | 2 | - | human |  |
|  | VIM-OXA 48 | *K. pneumoniae* | 2 | - | human |  |
| Iran | IMP+OXA-23-like | *A. baumannii* | 1 | 2019-2020 | environment | [67] |
| Thailand | OXA-23+NDM-1 | *A. baumannii* | 24 | 2013-2015 | human | [68] |
| Nepal | OXA-23+NDM-1 | *A. baumannii* | 6 | 2014-2015 | human | [69] |
| Uganda | KPC + IMP | *K. pneumoniae* | 1 | 2019 | human | [70] |
|  | IMP + OXA-48 | *K. pneumoniae* | 4 | 2019 | human |  |
|  | VIM + OXA-48 | *K. pneumoniae* | 7 | 2019 | human |  |
|  | VIM + NDM + OXA-48 | *K. pneumoniae* | 2 | 2019 | human |  |
|  | NDM + KPC + OXA-48 | *K. pneumoniae* | 2 | 2019 | human |  |
|  | IMP + NDM + OXA-48 | *K. pneumoniae* | 2 | 2019 | human |  |
|  | NDM + KPC + VIM + OXA-48 | *K. pneumoniae* | 1 | 2019 | human |  |
| Vietnam | NDM-1+OXA-23 | *A. baumannii* | 3 | 2011; 2013; 2014 | human | [71] |
| Iraq | IMP+OXA-48+NDM | *S. enteritidis* | 2 | 2018 | chicken meat | [72] |

**Table S2**. Strain information. Carbapenemases are underlined in bold. * If the gene sequence matches two gene variants with the same coverage and identity, both names of variants are given as ‘β-lactamase A or B’.

| label | species | β-lactamase profile | | | |
| --- | --- | --- | --- | --- | --- |
|  |  | A | B1 | C | D |
| K1N | *K. pneumoniae* | SHV-28 or 106*; CTX-M-15 | **NDM-1** | CMY-6 | **OXA-181** |
| K2N | *K. pneumoniae* | CTX-M-15; SHV-28 or 106*; TEM-1B; | **NDM-1** | CMY-6 | **OXA-181** |
| K3N | *K. pneumoniae* | CTX-M-15; SHV-28 or 106*; TEM-1B | **NDM-1** | CMY-6 | **OXA-181** |
| K4N | *K. pneumoniae* | TEM-1B; SHV-28 or 106*; CTX-M-15 | **NDM-1** |  | OXA-1; **OXA-232**; OXA-9 |
| K5N | *K. pneumoniae* | CTX-M-15; SHV-28 or 106* | **NDM-1** | CMY-6 | **OXA-181** |
| K1C | *K. pneumoniae* | CTX-M-15; TEM-1B; SHV-28 or 106* | **NDM-1** | CMY-6 | **OXA-181** |
| K6N | *K. pneumoniae* | SHV-182; CTX-M-15 | **NDM-5** |  | OXA-1 |
| K7N | *K. pneumoniae* | SHV-182 | **NDM-7; NDM-1** | CMY-6 | OXA-1 |
| K8N | *K. pneumoniae* | CTX-M-15; TEM-1C; SHV-38 | **NDM-7** |  |  |
| K9N | *K. pneumoniae* | SHV-38; TEM-1C; CTX-M-15 | **NDM-7** |  |  |
| S2E | *K. pneumoniae* |  | **NDM-7** |  |  |
| E1A | *K. quasipneumoniae* | LAP-2; SHV-12; OKP-A-1 | **NDM-1** |  |  |
| E1N | *E. coli* | TEM-1B; TEM-1A; CTX-M-15 | **NDM-1** | CMY-4 | OXA-2 |
| E2N | *E. coli* | CTX-M-15; TEM-1B; TEM-1A | **NDM-1** | CMY-6 | OXA-2 |
| E3N | *E. coli* | TEM-1A; CTX-M-15 | **NDM-1** |  | OXA-2 |
| E8N | *E. coli* | TEM-1B; CTX-M-15 | **NDM-5** |  | OXA-1 |
| E9N | *E. coli* |  | **NDM-5** | CMY-42 |  |
| E10N | *E. coli* | CTX-M-15 | **NDM-5** |  | OXA-1 |
| E11N | *E. coli* | CTX-M-15 | **NDM-7** | CMY-6 |  |
| C1A | *E. coli* | TEM-1B; CTX-M-15 | **NDM-4** | CMY-2 | OXA-1 |
| C5A | *C. sedlakii* | CTX-M-15; TEM-1B; SED1 | **NDM-1** | CMY-6 | OXA-1 |
| C3A | *E. hormaechei* | CTX-M-15; LAP-2 | **NDM-1** | ACT-5 | OXA-9 |
| E2A | *E. hormaechei* | GES-1 | **NDM-1** | ACT-16 | OXA-1 |
| E3A | *E. hormaechei* | CTX-M-15; TEM-1B | **NDM-1** | CMY-6; ACT-7 | OXA-1 |
| S1A | *S. marcescens* |  | **NDM-1** | SRT-1 or 2* |  |
| S2A | *S. marcescens* |  | **NDM-1** | SRT-1 or 2* |  |
| S3A | *S. marcescens* |  | **NDM-1** | SRT-1 or 2* |  |
| S4A | *S. marcescens* |  | **NDM-1** | SRT-1 or 2* |  |
| S5A | *S. marcescens* |  | **NDM-1** | SRT-1 or 2* |  |
| A2K | *A. nosocomialis* |  | **NDM-1** |  |  |
| A10K | *A. baumannii* | CARB-10 | **NDM-1** | ADC-25 | **OXA-98** |
| A11K | *A. baumannii* |  | **NDM-1** | ADC-25 | **OXA-203** |
| E4A | *E. hormaechei* | SHV-12 | **VIM-1; NDM-1** | ACT-16 |  |
| E5A | *E. hormaechei* | CTX-M-3; SHV-5 | **VIM-2** | ACT-7 |  |
| B0H | *P. aeruginosa* | VEB-1 | **VIM-2** | PAO | OXA-488 |
| B2H | *P. aeruginosa* |  | **VIM-2** | PAO | OXA-485 or 488*; OXA-10 |
| B3H | *P. aeruginosa* |  | **VIM-2** | PAO | OXA-485 or 488*; OXA-10 |
| B4H | *P. aeruginosa* |  | **VIM-28** |  |  |
| B5H | *P. aeruginosa* |  | **VIM-24** | PAO | OXA-10; OXA-488 |
| K2K | *K. pneumoniae* | TEM-1B; SHV-28 or 106*; **KPC-2**; CTX-M-15; VEB-1 |  |  | OXA-10 |
| K3K | *K. pneumoniae* | SHV-28 or 106*; TEM-1B; **KPC-2** |  |  |  |
| K4K | *K. pneumoniae* | SHV-28 or 106*; TEM-1B; **KPC-2** |  |  |  |
| K5K | *K. pneumoniae* | TEM-1B; SHV-28 or 106*; **KPC-2** |  |  |  |
| K2O | *K. pneumoniae* | CTX-M-15; TEM-1B; SHV-11 or 67* |  |  | **OXA-48** |
| K3O | *K. pneumoniae* | CTX-M-15; SHV-11 or 67*; TEM-1B |  |  | **OXA-48** |
| K1K | *K. pneumoniae* | SHV-28 or 106*; CTX-M-15; TEM-1B; VEB-1 |  |  | **OXA-48**; OXA-10 |
| K7O | *K. pneumoniae* | SHV-28 or 106* |  |  | **OXA-232**; OXA-1 |
| A1K | *A. baumannii* | CARB-2; PER-7 |  |  | **OXA-23; OXA-144** |
| A3K | *A. baumannii* | TEM-1D |  |  | **OXA-23; OXA-66** |
| A4K | *A. baumannii* | PER-7 |  |  | **OXA-23; OXA-64** |
| S3K | *S. maltophilia* |  |  |  |  |

**Table S3**. Susceptibility of parental strains, resistant mutants, and sensitive mutants. InC58 was at a fixed concentration of 4 mg/L.

| strain | label | MEM | MEM-InC58 |
| --- | --- | --- | --- |
| parental | E10N | 64 | 0.125 |
| resistant mutant | E10N-M1 | 256 | 2 |
| sensitive mutant | E10N-M1-R1 | 64 | 0.25 |
| sensitive mutant | E10N-M1-R2 | 64 | 0.25 |
| resistant mutant | E10N-M2 | 256 | 2 |
| sensitive mutant | E10N-M2-R1 | 64 | 0.25 |
| sensitive mutant | E10N-M2-R2 | 64 | 0.25 |
| parental | S2E | 64 | 0.5 |
| resistant mutant | S2E-M1 | >256 | 32 |
| sensitive mutant | S2E-M1-R1 | 128 | 1 |
| sensitive mutant | S2E-M1-R2 | 256 | 0.5 |
| resistant mutant | S2E-M2 | >256 | 32 |
| sensitive mutant | S2E-M2-R1 | 256 | 1 |
| parental | K8N | 64 | 0.5 |
| resistant mutant | K8N-M2 | >256 | 32 |
| sensitive mutant | K8N-M2-R1 | 128 | 0.5 |
| sensitive mutant | K8N-M2-R2 | 128 | 1 |
| resistant mutant | K8N-M9 | >256 | 32 |
| sensitive mutant | K8N-M9-R1 | 128 | 1 |
| sensitive mutant | K8N-M9-R2 | 128 | 0.5 |
| parental | K9N | 64 | 0.5 |
| resistant mutant | K9N-M2 | >256 | 32 |
| sensitive mutant | K9N-M2-R1 | 128 | 0.5 |
| sensitive mutant | K9N-M2-R2 | 128 | 0.5 |
| resistant mutant | K9N-M3 | >256 | 32 |
| sensitive mutant | K9N-M3-R1 | 128 | 0.5 |
| sensitive mutant | K9N-M3-R2 | 128 | 0.5 |

**Table S4**. Characterization of variant genes/products in resistant mutants, and their counterparts in corresponding sensitive mutants (highlighted in grey). *: stop code; fs: frameshift.

| Mutant | Reference  Sequence | Mutant  Sequence | Nucleotide  Position | Amino Acid  Position | Effect | Gene | Product |
| --- | --- | --- | --- | --- | --- | --- | --- |
| S4A-M1 | C | CTTCCTGCGCATGATGT | 432/645 | 144/214 | frameshift; Ala145fs | comR_2 | HTH-type transcriptional repressor ComR |
| S4A-M2 | C | CTTCCTGCGCATGATGT | 432/645 | 144/214 | frameshift; Ala145fs | comR_2 | HTH-type transcriptional repressor ComR |
| E10N-M1 | G | C | 884/1122 | 295/373 | missense; Arg295Pro | ompC | outer membrane porin C |
| E10N-M1-R1 | CG | TC | 883/1122 | 295/373 | missense; Arg295Ser | ompC | outer membrane porin C |
| E10N-M1-R2 | CG | TC | 883/1122 | 295/373 | missense; Arg295Ser | ompC | outer membrane porin C |
| E10N-M2 | G | C | 884/1122 | 295/373 | missense; Arg295Pro | ompC | outer membrane porin C |
| E10N-M2-R1 | CG | TC | 883/1122 | 295/373 | missense; Arg295Ser | ompC | outer membrane porin C |
| E10N-M2-R2 | CG | TC | 883/1122 | 295/373 | missense; Arg295Ser | ompC | outer membrane porin C |
| S2E-M1 | G | T | 378/783 | 126/260 | synonymous; Pro126Pro | argT | Lysine/arginine/ornithine-binding periplasmic protein |
| S2E-M1-R1 | G | T | 378/783 | 126/260 | synonymous; Pro126Pro | argT | Lysine/arginine/ornithine-binding periplasmic protein |
| S2E-M1-R2 | G | T | 378/783 | 126/260 | synonymous; Pro126Pro | argT | Lysine/arginine/ornithine-binding periplasmic protein |
| S2E-M1 | C | T | 370/783 | 124/260 | stop_gained; Gln124* | ompC | Outer membrane porin C |
| S2E-M1-R1 | CA | TT | 370/783 | 124/260 | missense; Gln124Leu | ompC | Outer membrane porin C |
| S2E-M1-R2 | C | T | 370/783 | 124/260 | stop_gained; Gln124* | ompC | Outer membrane porin C |
| S2E-M2 | G | T | 378/783 | 126/260 | synonymous; Pro126Pro | argT | Lysine/arginine/ornithine-binding periplasmic protein |
| S2E-M2-R1 | G | T | 378/783 | 126/260 | synonymous; Pro126Pro | argT | Lysine/arginine/ornithine-binding periplasmic protein |
| S2E-M2 | C | T | 370/783 | 124/260 | stop_gained; Gln124* | ompC | Outer membrane porin C |
| S2E-M2-R1 | C | T | 370/783 | 124/260 | stop_gained; Gln124* | ompC | Outer membrane porin C |
| S2E-M3 | - | - | - | - | - | - | - |
| K1N-M1 | T | G | 859/1104 | 287/367 | missense; Tyr287Asp | ompC | Outer membrane porin C |
| K1N-M1 | C | A | 894/1014 | 298/337 | synonymous; Gly298Gly | rssB | Regulator of RpoS |
| K1N-M1 | C | T | 600/606 | 200/201 | synonymous; Gly200Gly | - | Erse |
| K1N-M2 | - | - | - | - | - | - | - |
| K1N-M3 | - | - | - | - | - | - | - |
| K1N-M4 | - | - | - | - | - | - | - |
| K5N-M1 | A | T | 895/1104 | 299/367 | missense; Tyr299Asn | ompC | Outer membrane porin C |
| K5N-M2 | A | T | 895/1104 | 299/367 | missense; Tyr299Asn | ompC | Outer membrane porin C |
| K5N-M3 | A | T | 895/1104 | 299/367 | missense; Tyr299Asn | ompC | Outer membrane porin C |
| K8N-M1 | G | T | 232/1608 | 78/535 | missense; Gly78Trp | araB_1 | Ribulokinase |
| K8N-M1 | G | A | 373/513 | 125/170 | missense; Ala125Thr | moaB | Molybdenum cofactor biosynthesis protein B |
| K8N-M2 | G | T | 232/1608 | 78/535 | missense; Gly78Trp | araB_1 | Ribulokinase |
| K8N-M2-R1 | G | T | 232/1608 | 78/535 | missense; Gly78Trp | araB_1 | Ribulokinase |
| K8N-M2-R2 | G | T | 232/1608 | 78/535 | missense; Gly78Trp | araB_1 | Ribulokinase |
| K8N-M2 | G | A | 373/513 | 125/170 | missense; Ala125Thr | moaB | Molybdenum cofactor biosynthesis protein B |
| K8N-M2-R1 | G | A | 373/513 | 125/170 | missense; Ala125Thr | moaB | Molybdenum cofactor biosynthesis protein B |
| K8N-M2-R2 | G | A | 373/513 | 125/170 | missense; Ala125Thr | moaB | Molybdenum cofactor biosynthesis protein B |
| K8N-M3 | G | T | 232/1608 | 78/535 | missense; Gly78Trp | araB_1 | Ribulokinase |
| K8N-M3 | G | A | 373/513 | 125/170 | missense; Ala125Thr | moaB | Molybdenum cofactor biosynthesis protein B |
| K8N-M4 | G | T | 232/1608 | 78/535 | missense; Gly78Trp | araB_1 | Ribulokinase |
| K8N-M4 | G | A | 373/513 | 125/170 | missense; Ala125Thr | moaB | Molybdenum cofactor biosynthesis protein B |
| K8N-M5 | G | T | 232/1608 | 78/535 | missense; Gly78Trp | araB_1 | Ribulokinase |
| K8N-M5 | T | TA | 3/1095 | 1/364 | frameshift; Lys2fs | ompC | Outer membrane porin C |
| K8N-M5 | G | A | 373/513 | 125/170 | missense; Ala125Thr | moaB | Molybdenum cofactor biosynthesis protein B |
| K8N-M6 | G | T | 232/1608 | 78/535 | missense; Gly78Trp | araB_1 | Ribulokinase |
| K8N-M6 | T | TA | 3/1095 | 1/364 | frameshift; Lys2fs | ompC | Outer membrane porin C |
| K8N-M6 | G | A | 373/513 | 125/170 | missense; Ala125Thr | moaB | Molybdenum cofactor biosynthesis protein B |
| K8N-M7 | G | T | 232/1608 | 78/535 | missense; Gly78Trp | araB_1 | Ribulokinase |
| K8N-M7 | G | A | 373/513 | 125/170 | missense; Ala125Thr | moaB | Molybdenum cofactor biosynthesis protein B |
| K8N-M8 | - | - | - | - | - | - | - |
| K8N-M9 | G | T | 232/1608 | 78/535 | missense; Gly78Trp | araB_1 | Ribulokinase |
| K8N-M9-R1 | G | T | 232/1608 | 78/535 | missense; Gly78Trp | araB_1 | Ribulokinase |
| K8N-M9-R2 | G | T | 232/1608 | 78/535 | missense; Gly78Trp | araB_1 | Ribulokinase |
| K8N-M9 | G | A | 373/513 | 125/170 | missense; Ala125Thr | moaB | Molybdenum cofactor biosynthesis protein B |
| K8N-M9-R1 | G | A | 373/513 | 125/170 | missense; Ala125Thr | moaB | Molybdenum cofactor biosynthesis protein B |
| K8N-M9-R2 | G | A | 373/513 | 125/170 | missense; Ala125Thr | moaB | Molybdenum cofactor biosynthesis protein B |
| K8N-M10 | - | - | - | - | - | - | - |
| K8N-M11 | G | T | 232/1608 | 78/535 | missense; Gly78Trp | araB_1 | Ribulokinase |
| K8N-M11 | G | A | 373/513 | 125/170 | missense; Ala125Thr | moaB | Molybdenum cofactor biosynthesis protein B |
| K8N-M12 | G | T | 232/1608 | 78/535 | missense; Gly78Trp | araB_1 | Ribulokinase |
| K8N-M12 | G | A | 373/513 | 125/170 | missense; Ala125Thr | moaB | Molybdenum cofactor biosynthesis protein B |
| K9N-M1 | A | C | 1084/1095 | 362/364 | missense; Tyr362Asp | ompC | Outer membrane porin C |
| K9N-M2 | - | - | - | - | - | - | - |
| K9N-M2-R1 | - | - | - | - | - | - | - |
| K9N-M2-R2 | - | - | - | - | - | - | - |
| K9N-M3 | G | GT | 164/1095 | 55/364 | frameshift; Tyr56fs | ompC | Outer membrane porin C |
| K9N-M3-R1 | G | GT | 164/1095 | 55/364 | frameshift; Tyr56fs | ompC | Outer membrane porin C |
| K9N-M3-R1 | AG | A | 132/1095 | 44/364 | frameshift; Ser45fs | ompC | Outer membrane porin C |
| K9N-M3-R2 | G | GT | 164/1095 | 55/364 | frameshift; Tyr56fs | ompC | Outer membrane porin C |

**Table S5**. pIC50s of avibactam and InC58 towards various MBLs/SBLs. Errors for pIC50s are ±0.2 log fold. Data cited from published work [73]. NA: Not Applicable.

|  |  | Avibactam | InC58 |
| --- | --- | --- | --- |
| MBL | NDM-1 | NA | 9.5 |
|  | VIM-1 | NA | 9.1 |
|  | VIM-2 | NA | 9.2 |
|  | IMP-1 | NA | 7.2 |
| SBL | AmpC | 6.8 | NA |
|  | OXA-10 | >4.4 | NA |
|  | OXA-48 | 5.2 | NA |
|  | TEM-116 | 8.2 | NA |

**Fig. S1**. The global emergence of strains co-carrying SBL- and MBL-type carbapenemases. Major SBL-type carbapenemases (KPCs, OXA-23, and OXA-48/48-like) and MBL-type carbapenemases (NDMs, VIMs, and IMPs) are included. Data shown in the map includes a summary of results from an earlier study [74], supplemented with updated literature screening results (Table S1 in Appendix A).


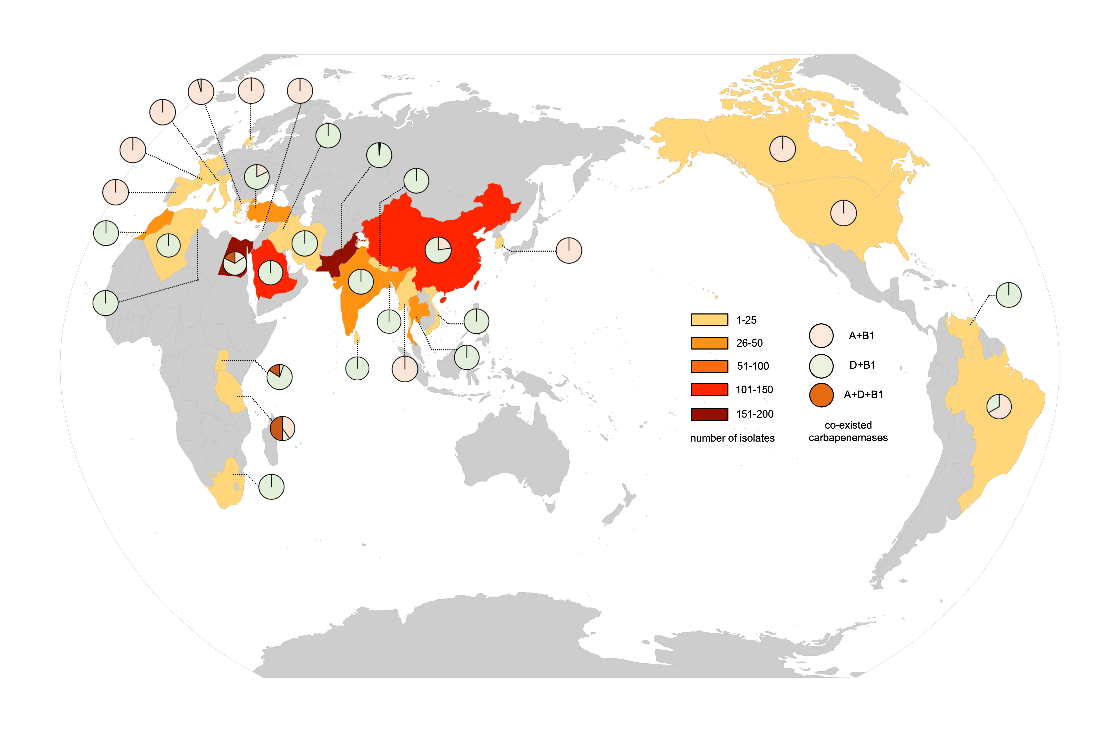


**Figure S2**. MICs of spontaneous resistant mutants and parental strains against varied concentrations of InC58 in combination with MEM.


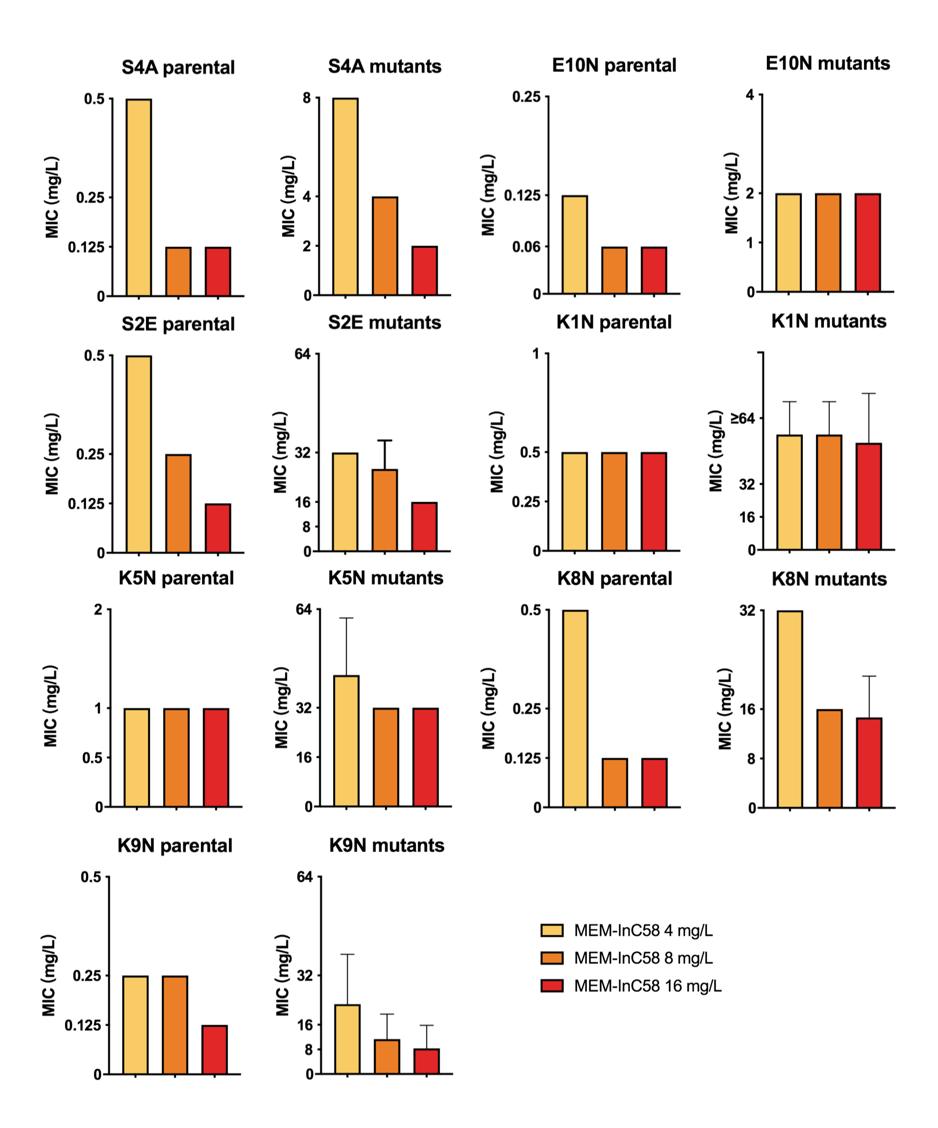


**Figure S3**. Amino acid sequence alignment of OmpC in *E. coli* that is resistant to MEM-InC58 (E10N-M1/M2) and its susceptible counterparts (E10N and E10N-M1/M2-R1/R2). The alignment was performed using Geneious Prime 2023.2.1.


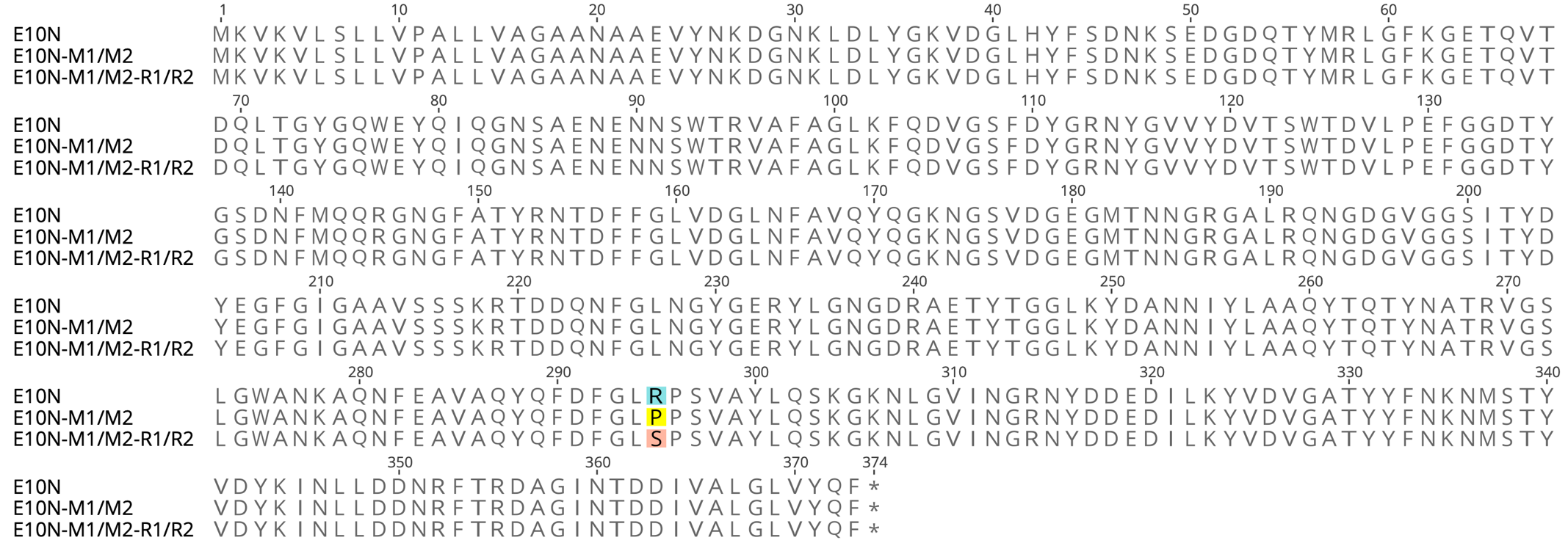


**Reference**

[1] Dang B, Zhang H, Li Z, Ma S, Xu Z. Coexistence of the *bla*_NDM-1_-carrying plasmid pWLK-NDM and the *bla*_KPC-2_-carrying plasmid pWLK-KPC in a *Raoultella ornithinolytica* isolate. *Sci Rep* 2020; **10**:2360.

[2] Sun Q, Dai Y, Chen J, Yu K, Wang Y, Zhang Y, et al. Coexistence of two *bla*_KPC-2_ genes in a *bla*_NDM-1_-carrying multidrug-resistant ST15 *Klebsiella pneumoniae* isolate recovered from cerebrospinal fluid in China. *J Glob Antimicrob Resist* 2022; **29**:232-5.

[3] Feng J, Qiu Y, Yin Z, Chen W, Yang H, Yang W, et al. Coexistence of a novel KPC-2-encoding MDR plasmid and an NDM-1-encoding pNDM-HN380-like plasmid in a clinical isolate of *Citrobacter freundii*. *J Antimicrob Chemother* 2015; **70**: 2987-91.

[4] Fu L, Wang S, Zhang Z, Yan X, Yang X, Zhang L, et al. Co-carrying of KPC-2, NDM-5, CTX-M-3 and CTX-M-65 in three plasmids with serotype O89: H10 *Escherichia coli* strain belonging to the ST2 clone in China. *Microb Pathog* 2019; **128**: 1-6.

[5] Zhao Y, Tang N, Jia R, Hu J, Liu W, Sun Y, et al. Co-existence of the carbapenem resistance genes *bla*_KPC-2_ and *bla*_NDM-1_ in a *Raoultella planticola* isolate in China. *J Glob Antimicrob Resist* 2020; **23**: 327-328.

[6] Zhang Y, Gu D, Yang X, Wu Y, Liu C, Shen Z, et al. Emergence and Genomic Characterization of a KPC-2-, NDM-1-, and IMP-4-Producing *Klebsiella michiganensis* Isolate. *Front Microbiol* 2022; **12**: 762509.

[7] Wu WJ, Feng Y, Carattoli A, Zong ZY. Characterization of an *Enterobacter cloacae* Strain Producing both KPC and NDM Carbapenemases by Whole-Genome Sequencing. *Antimicrob Agents Chemother* 2015; **59**: 6625-8.

[8] Li X, Wang W, Jin X, Zhang X, Zou X, Ma Q, et al. Emergence of Plasmids Co-Harboring Carbapenem Resistance Genes and tmexCD2-toprJ2 in Sequence Type 11 Carbapenem Resistant *Klebsiella pneumoniae* Strains. *Front Cell Infect Microbiol* 2022; **12**: 902774.

[9] Hu L, Liu Y, Deng L, Zhong Q, Hang Y, Wang Z, et al. Outbreak by Ventilator-Associated ST11 *K. pneumoniae* with Co-production of CTX-M-24 and KPC-2 in a SICU of a Tertiary Teaching Hospital in Central China. *Front Microbiol* 2016; **7**: 1190.

[10] Wu W, Espedido B, Feng Y, Zong Z. *Citrobacter freundii* carrying *bla*_KPC-2_ and *bla*_NDM-1_: characterization by whole genome sequencing. *Sci Rep* 2016; **6**: 30670.

[11] Ouyang J, Sun F, Zhou D, Feng J, Zhan Z, Xiong Z, et al. Comparative genomics of five different resistance plasmids coexisting in a clinical multi-drug resistant *Citrobacter freundii* isolate. *Infect Drug Resist* 2018; **11**: 1447-1460.

[12] Tang Y, Zhou Y, Meng C, Huang Y, Jiang X. Co-occurrence of a novel VIM-1 and FosA3-encoding multidrug-resistant plasmid and a KPC-2-encoding pKP048-like plasmid in a clinical isolate of *Klebsiella pneumoniae* sequence type 11. *Infect Genet Evol* 2020; **85**: 104479.

[13] Wei Z, Yu T, Qi Y, Ji S, Shen P, Yu Y, et al. Coexistence of plasmid-mediated KPC-2 and IMP-4 carbapenemases in isolates of *Klebsiella pneumoniae* from China. *J Antimicrob Chemother* 2011; **66**: 2670-1.

[14] Zheng B, Zhang J, Ji J, Fang Y, Shen P, Ying C, et al. Emergence of *Raoultella ornithinolytica* coproducing IMP-4 and KPC-2 carbapenemases in China. *Antimicrob Agents Chemother* 2015; **59**: 7086-9.

[15] Hu L, Zhong Q, Shang Y, Wang H, Ning C, Li Y, et al. The prevalence of carbapenemase genes and plasmid-mediated quinolone resistance determinants in carbapenem-resistant Enterobacteriaceae from five teaching hospitals in central China. *Epidemiol Infect* 2014; **142**: 1972-7.

[16] Wang Y, Cao W, Zhu X, Chen Z, Li L, Zhang B, et al. Characterization of a novel *Klebsiella pneumoniae* sequence type 476 carrying both *bla* _KPC-2_ and *bla* _IMP-4_. *Eur J Clin Microbiol Infect Dis* 2012; **31**: 1867-72.

[17] Wang D, Zhu J, Zhou K, Chen J, Yin Z, Feng J, et al. Genetic characterization of novel class 1 Integrons In0, In1069 and In1287 to In1290, and the inference of In1069-associated integron evolution in *Enterobacteriaceae*. *Antimicrob Resist Infect Control* 2017; **6**: 84.

[18] Xie L, Dou Y, Zhou K, Chen Y, Han L, Guo X, et al. Coexistence of *bla*_OXA-48_ and Truncated *bla*_NDM-1_ on Different Plasmids in a *Klebsiella pneumoniae* Isolate in China. *Front Microbiol* 2017; **8**: 133.

[19] Zhang B, Hu R, Liang Q, Liang S, Li Q, Bai J, et al. Comparison of Two Distinct Subpopulations of *Klebsiella pneumoniae* ST16 Co-Occurring in a Single Patient. *Microbiol Spectr* 2022; **10**: e0262421.

[20] Sun X, Liu B, Chen Y, Huang H, Wang G, Li F, et al. Molecular characterization of Ambler class A to D β-lactamases, ISAba1, and integrons reveals multidrug-resistant *Acinetobacter* spp*.* isolates in northeastern China. *J Chemother* 2016; **28**: 469-475.

[21] Patil S, Chen H, Guo C, Zhang X, Ren PG, Francisco NM, et al. Emergence of *Klebsiella pneumoniae* ST307 Co-Producing CTX-M with SHV and KPC from Paediatric Patients at Shenzhen Children's Hospital, China. *Infect Drug Resist* 2021; **14**: 3581-3588.

[22] Bail L, Ito CAS, Arend L, Pilonetto M, Nogueira KDS, Tuon FF. Distribution of genes encoding 16S rRNA methyltransferase in plazomicin-nonsusceptible carbapenemase-producing *Enterobacterales* in Brazil. *Diagn Microbiol Infect Dis* 2021; **99**: 115239.

[23] Flores C, Bianco K, de Filippis I, Clementino MM, Romao C. Genetic Relatedness of NDM-Producing *Klebsiella pneumoniae* Co-Occurring VIM, KPC, and OXA-48 Enzymes from Surveillance Cultures from an Intensive Care Unit. *Microb Drug Resist* 2020; **26**: 1219-1226.

[24] Wasfi R, Rasslan F, Hassan SS, Ashour HM, Abd El-Rahman OA. Co-Existence of Carbapenemase-Encoding Genes in *Acinetobacter baumannii* from Cancer Patients. *Infect Dis Ther* 2021; **10**: 291-305.

[25] El-Badawy MF, El-Far SW, Althobaiti SS, Abou-Elazm FI, Shohayeb MM. The First Egyptian Report Showing the Co-Existence of *bla*_NDM-25_, *bla*_OXA-23_, *bla*_OXA-181_, and *bla*_GEs-1_ Among Carbapenem-Resistant *K. pneumoniae* Clinical Isolates Genotyped by BOX-PCR. *Infect Drug Resist* 2020; **13**: 1237-1250.

[26] El-Mahallawy HA, El Swify M, Hak AA, Zafer MM. Increasing trends of colistin resistance in patients at high-risk of carbapenem-resistant *Enterobacteriaceae*. *Ann Med* 2022; **54**: 1-9.

[27] Khalil MAF, Ahmed FA, Elkhateeb AF, Mahmoud EE, Ahmed MI, Ahmed RI, et al. Virulence Characteristics of Biofilm-Forming *Acinetobacter baumannii* in Clinical Isolates Using a *Galleria mellonella* Model. *Microorganisms* 2021; **9**: 2365.

[28] Khalifa HO, Soliman AM, Ahmed AM, Shimamoto T, Hara T, Ikeda M, et al. High Carbapenem Resistance in Clinical Gram-Negative Pathogens Isolated in Egypt. *Microb Drug Resist* 2017; **23**: 838-844.

[29] ElMahallawy H, Zafer MM, Al-Agamy M, Amin MA, Mersal MM, Booq RY, et al. Dissemination of ST101 *bla*_OXA-48_ producing *Klebsiella pneumoniae* at tertiary care setting. *J Infect Dev Ctries* 2018; **12**: 422-428.

[30] Ragheb SM, Tawfick MM, El-Kholy AA, Abdulall AK. Phenotypic and Genotypic Features of *Klebsiella pneumoniae* Harboring Carbapenemases in Egypt: OXA-48-Like Carbapenemases as an Investigated Model. *Antibiotics (Basel)* 2020; **9**: 852.

[31] Ghaith DM, Zafer MM, Said HM, Elanwary S, Elsaban S, Al-Agamy MH, et al. Genetic diversity of carbapenem-resistant *Klebsiella Pneumoniae* causing neonatal sepsis in intensive care unit, Cairo, Egypt. *Eur J Clin Microbiol Infect Dis* 2020; **39**: 583-591.

[32] Abdelaziz NA. Phenotype-genotype correlations among carbapenem-resistant *Enterobacterales* recovered from four Egyptian hospitals with the report of SPM carbapenemase. *Antimicrob Resist Infect Control* 2022; **11**: 13.

[33] Benmahmod AB, Said HS, Ibrahim RH. Prevalence and Mechanisms of Carbapenem Resistance Among *Acinetobacter baumannii* Clinical Isolates in Egypt. *Microb Drug Resist* 2019; **25**: 480-488.

[34] Gomez-Gamboa L, Barrios-Camacho H, Duran-Bedolla J, Sanchez-Perez A, Reyna-Flores F, Perozo-Mena A, et al. Molecular and genetic characterization of carbapenemase-producing bacteria in Venezuela. *J Chemother* 2019; **31**: 349-353.

[35] Haider MH, McHugh TD, Roulston K, Arruda LB, Sadouki Z, Riaz S. Detection of carbapenemases *bla*_OXA48_-*bla*_KPC_-*bla*_NDM_-bla_VIM_ and extended-spectrum-β-lactamase *bla*_OXA1_-*bla*_SHV_-*bla*_TEM_ genes in Gram-negative bacterial isolates from ICU burns patients. *Ann Clin Microbiol Antimicrob* 2022; **21**: 18.

[36] Sattar H, Toleman M, Nahid F, Zahra R. Co-existence of *bla*_NDM-1_ and *bla*_KPC-2_ in clinical isolates of *Klebsiella pneumoniae* from Pakistan. *J Chemother* 2016; **28**: 346-9.

[37] Gondal AJ, Saleem S, Jahan S, Choudhry N, Yasmin N. Novel Carbapenem-Resistant *Klebsiella pneumoniae* ST147 Coharboring *bla* _NDM-1_, *bla* _OXA-48_ and Extended-Spectrum β-Lactamases from Pakistan. *Infect Drug Resist* 2020; **13**: 2105-2115.

[38] Ejaz H, Qamar MU, Junaid K, Younas S, Taj Z, Bukhari SNA, et al. The Molecular Detection of Class B and Class D Carbapenemases in Clinical Strains of *Acinetobacter calcoaceticus-baumannii* Complex: The High Burden of Antibiotic Resistance and the Co-Existence of Carbapenemase Genes. *Antibiotics (Basel)* 2022; **11**: 1168.

[39] Sands K, Carvalho MJ, Portal E, Thomson K, Dyer C, Akpulu C, et al. Characterization of antimicrobial-resistant Gram-negative bacteria that cause neonatal sepsis in seven low- and middle-income countries. *Nature microbiology* 2021; **6**: 512-523.

[40] Naha S, Sands K, Mukherjee S, Saha B, Dutta S, Basu S. OXA-181-Like Carbapenemases in *Klebsiella pneumoniae* ST14, ST15, ST23, ST48, and ST231 from Septicemic Neonates: Coexistence with NDM-5, Resistome, Transmissibility, and Genome Diversity. *mSphere* 2021; **6**: e01156-20.

[41] Paul D, Dhar D, Chakravarty A, Bhattacharjee A. Transcriptional Analysis of IncF_repB_-Mediated *blaOXA-48*-Positive Plasmid Characterized from *Escherichia coli* ST448. *Microb Drug Resist* 2021; **27**: 596-601.

[42] Singh S, Pathak A, Rahman M, Singh A, Nag S, Sahu C, et al. Genetic Characterisation of Colistin Resistant *Klebsiella pneumoniae* Clinical Isolates From North India. *Front Cell Infect Microbiol* 2021; **11**: 666030.

[43] Karthikeyan K, Thirunarayan MA, Krishnan P. Coexistence of *bla*_OXA-23_ with *bla*_NDM-1_ and armA in clinical isolates of *Acinetobacter baumannii* from India. *J Antimicrob Chemother* 2010; **65**: 2253-4.

[44] Vamsi SK, Moorthy RS, Hemiliamma MN, Reddy RBC, Chanderakant DJ, Sirikonda S. Phenotypic and genotypic detection of carbapenemase production among gram negative bacteria isolated from hospital acquired infections. *Saudi Med J* 2022; **43**: 236-243.

[45] Gajamer VR, Bhattacharjee A, Paul D, Ingti B, Sarkar A, Kapil J, et al. High prevalence of carbapenemase, AmpC β-lactamase and aminoglycoside resistance genes in extended-spectrum β-lactamase-positive uropathogens from Northern India. *J Glob Antimicrob Resist* 2020; **20**: 197-203.

[46] Rakhi NN, Alam A, Sultana M, Rahaman MM, Hossain MA. Diversity of carbapenemases in clinical isolates: The emergence of *bla*_VIM-5_ in Bangladesh. *J Infect Chemother* 2019; **25**: 444-451.

[47] Jahan MI, Rahaman MM, Hossain MA, Sultana M. Draft genome sequence of a carbapenem-resistant clinical *Acinetobacter baumannii* revealing co-existence of four classes of beta-lactamases. *J Glob Antimicrob Resist* 2021; **27**: 329-331.

[48] Ciftci E, Sesli Cetin E, Us E, Haydar Kutlu H, Cicioglu Aridogan B. Investigation of Carbapenem resistance mechanisms in *Klebsiella pneumoniae* by using phenotypic tests and a molecular assay. *J Infect Dev Ctries* 2019; **13**: 992-1000.

[49] Iraz M, Özad Düzgün A, Sandallı C, Doymaz MZ, Akkoyunlu Y, Saral A, et al. Distribution of β-lactamase genes among carbapenem-resistant *Klebsiella pneumoniae* strains isolated from patients in Turkey. *Ann Lab Med* 2015; **35**: 595-601.

[50] Copur Cicek A, Erturk A, Ejder N, Rakici E, Kostakoglu U, Esen Yildiz I, et al. Screening of Antimicrobial Resistance Genes and Epidemiological Features in Hospital and Community-Associated Carbapenem-Resistant *Pseudomonas aeruginosa* Infections. *Infect Drug Resist* 2021; **14**: 1517-1526.

[51] Kutlu HH, Us E, Tekeli A. Investigation of Carbapenemase Genes and Molecular Epidemiology of *Enterobacteriaceae* Strains Isolated between 2010-2014 in a University Hospital. *Mikrobiyol Bul* 2018; **52**: 1-12.

[52] Vatansever C, Menekse S, Dogan O, Gucer LS, Ozer B, Ergonul O, et al. Co-existence of OXA-48 and NDM-1 in colistin resistant *Pseudomonas aeruginosa* ST235. *Emerg Microbes Infect* 2020; **9**: 152-154.

[53] El Kettani A, Maaloum F, Nzoyikorera N, Khalis M, Katfy K, Belabbes H, et al. Evaluation of the Performances of the Rapid Test RESIST-5 O.O.K.N.V Used for the Detection of Carbapenemases-Producing *Enterobacterales*. *Antibiotics (Basel)* 2021; **10**: 953.

[54] Uwingabiye J, Lemnouer A, Roca I, Alouane T, Frikh M, Belefquih B, et al. Clonal diversity and detection of carbapenem resistance encoding genes among multidrug-resistant *Acinetobacter baumannii* isolates recovered from patients and environment in two intensive care units in a Moroccan hospital. *Antimicrob Resist Infect Control* 2017; **6**: 99.

[55] Benaissa E, Abassour T, Belouad E, Maleb A, Elouennass M. Characterization of imipenem-resistant *Acinetobacter baumannii* and *Pseudomonas aeruginosa* clinical isolates in a Moroccan hospital. *Acta Microbiol Immunol Hung* 2022; **69**: 118-126.

[56] Hernandez-Garcia M, Perez-Viso B, Carmen Turrientes M, Diaz-Agero C, Lopez-Fresnena N, Bonten M, et al. Characterization of carbapenemase-producing *Enterobacteriaceae* from colonized patients in a university hospital in Madrid, Spain, during the R-GNOSIS project depicts increased clonal diversity over time with maintenance of high-risk clones. *J Antimicrob Chemother* 2018; **73**: 3039-3043.

[57] Perera V, de Silva S, Jayatilleke K, de Silva N, Aydin A, Enne V, et al. Antimicrobial Resistance Genes, Virulence Genes, and Associated Mobile Genetic Elements of Eight Multidrug-Resistant *Enterobacterales* Isolated from Hospital-Acquired Urinary Tract Infections in Sri Lanka. *Microb Drug Resist* 2022; **28**: 882-892.

[58] Ramoul A, Loucif L, Bakour S, Amiri S, Dekhil M, Rolain JM. Co-occurrence of *bla*_NDM-1_ with *bla*_OXA-23_ or *bla*_OXA-58_ in clinical multidrug-resistant *Acinetobacter baumannii* isolates in Algeria. *J Glob Antimicrob Resist* 2016; **6**: 136-141.

[59] Ben Sallem R, Laribi B, Arfaoui A, Ben Khelifa Melki S, Ouzari HI, Ben Slama K, et al. Co-occurrence of genes encoding carbapenemase, ESBL, pAmpC and non-β-Lactam resistance among *Klebsiella pneumonia* and *E. coli* clinical isolates in Tunisia. *Lett Appl Microbiol* 2022; **74**: 729-740.

[60] Al-Sultan AA, Evans BA, Aboulmagd E, Al-Qahtani AA, Bohol MF, Al-Ahdal MN, et al. Dissemination of multiple carbapenem-resistant clones of *Acinetobacter baumannii* in the Eastern District of Saudi Arabia. *Front Microbiol* 2015; **6**: 634.

[61] Memish ZA, Assiri A, Almasri M, Roshdy H, Hathout H, Kaase M, et al. Molecular Characterization of Carbapenemase Production Among Gram-Negative Bacteria in Saudi Arabia. *Microbial Drug Resistance* 2015; **21**: 307-14.

[62] Shah MW, Yasir M, Farman M, Jiman-Fatani AA, Almasaudi SB, Alawi M, et al. Antimicrobial Susceptibility and Molecular Characterization of Clinical Strains of *Acinetobacter baumannii* in Western Saudi Arabia. *Microb Drug Resist* 2019; **25**: 1297-1305.

[63] Poulou A, Grivakou E, Vrioni G, Koumaki V, Pittaras T, Pournaras S, et al. Modified CLSI extended-spectrum β-lactamase (ESBL) confirmatory test for phenotypic detection of ESBLs among *Enterobacteriaceae* producing various β-lactamases. *J Clin Microbiol* 2014; **52**: 1483-9.

[64] Silago V, Mruma EC, Msemwa B, Mtemisika CI, Phillip S, Ndagula RA, et al. Predominance of *Acinetobacter* spp., Harboring the *bla*_IMP_ Gene, Contaminating the Hospital Environment in a Tertiary Hospital in Mwanza, Tanzania: A Cross-Sectional Laboratory-Based Study. *Pathogens* 2022; **11**: 63.

[65] Anane YA, Apalata T, Vasaikar S, Okuthe GE, Songca S. Molecular Detection of Carbapenemase-Encoding Genes in Multidrug-Resistant *Acinetobacter baumannii* Clinical Isolates in South Africa. *Int J Microbiol* 2020; **2020**: 7380740.

[66] Mmatli M, Leshaba TMS, Skosana LB, Mbelle NM, Sekyere JO. Molecular Screening of Clinical Multidrug-Resistant Gram-Negative Bacteria Shows Endemicity of Carbapenemases, Coexistence of Multiple Carbapenemases, and Rarity of *mcr* in South Africa. *Microb Drug Resist* 2022; **28**: 1028-1036.

[67] Yousefi Nojookambari N, Sadredinamin M, Dehbanipour R, Ghalavand Z, Eslami G, Vaezjalali M, et al. Prevalence of β-lactamase-encoding genes and molecular typing of *Acinetobacter baumannii* isolates carrying carbapenemase OXA-24 in children. *Ann Clin Microbiol Antimicrob* 2021; **20**: 75.

[68] Leungtongkam U, Thummeepak R, Wongprachan S, Thongsuk P, Kitti T, Ketwong K, et al. Dissemination of *bla*_OXA-23_, *bla*_OXA-24_, *bla*_OXA-58_, and *bla*_NDM-1_ Genes of *Acinetobacter baumannii* Isolates from Four Tertiary Hospitals in Thailand. *Microb Drug Resist* 2018; **24**: 55-62.

[69] Joshi PR, Acharya M, Kakshapati T, Leungtongkam U, Thummeepak R, Sitthisak S. Co-existence of *bla*_OXA-23_ and *bla*_NDM-1_ genes of *Acinetobacter baumannii* isolated from Nepal: antimicrobial resistance and clinical significance. *Antimicrob Resist Infect Control* 2017; **6**: 21.

[70] Ssekatawa K, Byarugaba DK, Nakavuma JL, Kato CD, Ejobi F, Tweyongyere R, et al. Prevalence of pathogenic *Klebsiella pneumoniae* based on PCR capsular typing harbouring carbapenemases encoding genes in Uganda tertiary hospitals. *Antimicrob Resist Infect Control* 2021; **10**: 57.

[71] Tran DN, Tran HH, Matsui M, Suzuki M, Suzuki S, Shibayama K, et al. Emergence of New Delhi metallo-beta-lactamase 1 and other carbapenemase-producing *Acinetobacter calcoaceticus-baumannii* complex among patients in hospitals in Ha Noi, Viet Nam. *Eur J Clin Microbiol Infect Dis* 2017; **36**: 219-225.

[72] Kanaan MHG, Khalil ZK, Khashan HT, Ghasemian A. Occurrence of virulence factors and carbapenemase genes in Salmonella enterica serovar Enteritidis isolated from chicken meat and egg samples in Iraq. BMC Microbiol 2022; 22: 279.

[73] Brem J, Panduwawala T, Hansen JU, Hewitt J, Liepins E, Donets P, et al. Imitation of β-lactam binding enables broad-spectrum metallo-β-lactamase inhibitors. Nat Chem 2022; 14:15-24.

[74] Carvalho MJ, Sands K, Thomson K, Portal E, Mathias J, Milton R, et al.; BARNARDS Group. Antibiotic resistance genes in the gut microbiota of mothers and linked neonates with or without sepsis from low- and middle-income countries. Nat Microbiol 2022;7(9):1337–47.
